# Supplementary material for: Overcoming resistance to anabolic SARM therapy in experimental cancer cachexia with an HDAC inhibitor
Source: EMBO Mol Med. 2020 Jan 13;12(2):e9910. doi: 10.15252/emmm.201809910 (PMC7005646; doi:10.15252/emmm.201809910)

Appendix for:

Overcoming Resistance to Anabolic SARM Therapy in Experimental Cancer Cachexia with an HDAC Inhibitor

Sophia G. Liva[1,*], Yu-Chou Tseng[2,*], Anees M. Dauki[1], Michael G. Sovic[1], Trang Vu[1], Sally E. Henderson[3], Yi-Chiu Kuo[2], Jason A. Benedict[4], Xiaoli Zhang[4], Bryan C. Remaily[1], Samuel K. Kulp[1], Moray Campbell[1], Tanios Bekaii-Saab[5], Mitchell A. Phelps[1], Ching-Shih Chen[2,6] and Christopher C. Coss[1]

[1] Division of Pharmaceutics and Pharmaceutical Chemistry, College of Pharmacy, The Ohio State University, Columbus, OH
[2] Division of Medicinal Chemistry and Pharmacognosy, College of Pharmacy, The Ohio State University, Columbus, OH
[3] Department of Veterinary Biosciences, College of Veterinary Medicine, Ohio State University, Columbus OH
[4] Center for Biostatistics, Department of Biomedical Informatics, The Ohio State University, Columbus, OH
[5] Mayo Clinic Cancer Center, Phoenix, AZ
[6] Department of Medical Research, China Medical University Hospital, China Medical University, Taichung 40447, Taiwan

*Equally contributing authors

Corresponding Author:
Christopher C. Coss, PhD
Mailing Address: The Ohio State University College of Pharmacy, 500 W.12th Ave, Columbus OH, 43210
Phone:614-688-1309
Fax: (614) 292-7766
E-mail:coss.16@osu.edu

Running title: SARM plus HDAC inhibitor for cancer cachexia

Table of Contents

Appendix Figure S1. 4

Appendix Figure S2. 4

Appendix Figure S3. 4

Appendix Figure S4. 4

Appendix Figure S5. 5

Appendix Figure S6. 5

Appendix Figure S7. 5

Appendix Figure S8. 5

Appendix Figure S9. 5

Appendix Figure S10. 5

Appendix Figure S11. 6

Appendix Figure S12. 6

Appendix Figure S13. 6

Appendix Figure S14. 6

Appendix Figure S15. 6

Appendix Tables 7

Appendix Table S1A-D (related to Figure 1) 7

Appendix Table S2A-D (related to Figure 2) 8

Appendix Table S3A-D (related to Figure 3) 9

Appendix Table S4A-D (related to Figure 4) 10

Appendix Table S5A-B (related to Figure 5)....11

Appendix Table 6A-B (related to Figure 6) 12

Appendix Table 7 (related to Figure 7)....13

Appendix Table 8 (related to Figure 8)....14

Appendix Table 9 (related to Figure EV1)....15

Appendix Table 10 (related to Figure EV2)....16

Appendix Table 11 (related to Figure EV3)....17

Appendix Table 12 (related to Figure EV4)....18

Appendix Table S13. Serum Cytokine Panel – Complete Results. 19

Appendix Table S14. Primer sequences. 21

Appendix Table S15. Post-alignment quality control metrics for RNA-seq dataset. 22

Appendix References..23

Appendix Figures

Appendix Figure S1.

A) Non-compartmental analysis of single dose AR-42 pharmacokinetics in mouse. Parameters were derived as outlined in the *Materials and Methods* section. **B)** AR-42 dose-response. Starting six days after C-26 cell injection, animals received vehicle or AR-42 orally at 1 (n=8), 3 (n=8), 10 (n=13) or 20 mg/kg (n=6) daily or 50 mg/kg (n=5) every other day for 13 days. Individual animal gastrocnemius weights are presented as a percentage of tumor-free control muscle weight. Dashed reference line (100%) represents the mean tumor-free gastrocnemius mass, solid line represents non-linear fit of dose-response data. **C)** AR-42's *in vitro* human HDAC isoform inhibition profile. HDAC activity of recombinant human HDAC enzymes was determined in the presence of 1 μM AR-42 as outlined in the *Materials and Methods*.

Appendix Figure S2.

Study 1 (male; n=20) and Study 3 (female; n=43) C-26 tumor volumes. Terminal tumor volume comparisons between Study 1 and Study 3. *Statistics:* ns, no significant between-study differences in tumor volumes. Student's t-test of combined tumor volumes (Study 1 versus Study 3). ns, no significant differences among treatment groups within each study, one-way ANOVA followed by Tukey's multiple comparison test. Individual p-values displayed in tables.

Appendix Figure S3.

Study 4, tumor-bearing male mice receiving AR-42 (10 mg/kg, oral gavage; n=9), TFM-4AS-1 (10 mg/kg, subcutaneous; n=10), Combination of AR-42 and DHT (10 mg/kg oral gavage and 3 mg/kg subcutaneous, respectively; n=10), Combination of AR-42 and TFM-4AS-1 (10 mg/kg, both, n=9) or Vehicle (n=6) and tumor-free mice receiving vehicle (n=6) were treated daily for 12 days starting 6 days post-injection of C-26 cells. **A)** Tumor volume comparisons between Day 8 and Day 16 post-C-26 cell injection. *Statistics:* Individual p-values displayed in tables; ns, no significant differences; one-way ANOVA followed by Tukey's multiple comparison test. **B)** Androgen receptor western blot of C-26 and LLC tumor tissue and associated source cells. Mouse prostate tissue was analyzed as a positive control. **C)** C-26 cell viability was determined after 48 hours of treatment with DHT or GTx-024 at concentrations of up to 10 μM. Data in panels A and C are presented as means ± SD.

Appendix Figure S4.

Study 4**,** Terminal epididymal fat pad mass from mice in Study 4 treated as described in Appendix Figure S7. *Statistics:* V, T, A indicate statistically significant differences versus tumor-bearing vehicle-treated, tumor-bearing TFM-4AS-1-treated and tumor-bearing AR-42-treated groups, respectively. Individual p-values displayed in tables, one-way ANOVA followed by Tukey's multiple comparison test. Data are presented as means ± SD.

Appendix Figure S5.
Western blot analysis of AR in gastrocnemius muscles from representative mice in Study 1. Black circle – tumor-free, blue square – tumor-free/GTx-024, black triangle – tumor-bearing, blue diamond – tumor/GTx-024, red triangle – tumor/AR-42, green circle – tumor/combo.

Appendix Figure S6.
A) Genes differentially regulated in gastrocnemius muscle by 50 mg/kg AR-42 treatment relative to C-26 tumor-bearing vehicle-treated controls from Tseng *et al*. intersected with genes differentially regulated in quadriceps muscle from both severe and moderately wasting C-26 tumor-bearing mice relative to tumor-free controls from Bonetto *et al.* **B)** Canonical pathway analysis using GSEA of the 147 overlapping genes from **A**.

Appendix Figure S7.
Western blot analysis of phospho(p)STAT3 in gastrocnemius muscles from representative mice treated in Study 1. This data is a replicate blot generated from the samples used in Figure 6B. tSTAT3, total STAT3.

Appendix Figure S8.
Principle Component Analysis plots of Study 1 RNA-seq samples utilized for subsequent analyses.

Appendix Figure S9.
Standard volcano plots from RNA-seq analyses of Study 1 gastrocnemius muscles showing **A)** DEGs for tumor-bearing (cachectic) GTx-024-treated (Cx+GTx-024) mice versus tumor-free controls (colored red), and **B)** DEGs for tumor-bearing AR-42-treated (Cx+AR-42) mice versus tumor-free controls (colored red). Log2-transformed fold change (FC) in expression is plotted on the x-axis and -log10 transformed Benjamini-Hochburg adjusted p-values are plotted on the y-axis.

Appendix Figure S10.
Enrichment plots of the STAT3 gene set for each treatment group versus tumor-bearing control comparisons. Tumor-free control (black checkered), GTx-024-treated tumor-free (blue checkered), GTx-024-treated tumor-bearing (blue), AR-42-treated tumor-bearing (red) and Combination-treated tumor-bearing (green).

Appendix Figure S11.

Enrichment plots of the ATF-1 gene set for each treatment group versus tumor-bearing control comparisons. Tumor-free control (black checkered), GTx-024-treated tumor-free (blue checkered), GTx-024-treated tumor-bearing (blue), AR-42-treated tumor-bearing (red) and Combination-treated tumor-bearing (green).

Appendix Figure S12.

A) Genes differentially regulated 4-fold or greater in gastrocnemius muscle by 50 mg/kg AR-42 treatment relative to tumor-bearing vehicle-treated controls from Tseng *et al*. intersected with genes differentially regulated 2-fold or greater in gastrocnemius muscle by 10 mg/kg AR-42 treatment relative to tumor-bearing controls from Study 1. **B)** Scatter plot of the 209 overlapping genes identified in **(A)**. **C)** Canonical pathway analysis using the 147 overlapping genes identified in **(A)**.

Appendix Figure S13.

Standard volcano plot from RNA-seq analyses of Study 1 gastrocnemius muscles showing DEGs for GTx-024-treated tumor-free controls versus vehicle-treated tumor-free controls (colored red). Log2-transformed fold change (FC) in expression is plotted on the x-axis and -log10 transformed Benjamini-Hochburg adjusted p-values are plotted on the y-axis.

Appendix Figure S14.

Overlapping genes among leading-edge subsets from the enrichment analysis of the *Ctnnb1* gene set presented in Figure 8 were identified. The comparisons performed were among GTx-024-treated tumor-free mice (GTx-024, Tumor Free; GTx), tumor-bearing mice (Cachexia; Cx) and **A)** GTx-024-treated tumor-bearing mice (GTx-024, Tumor Bearing; GTB), **B)** AR-42-treated tumor-bearing mice (AR-42, Tumor bearing; ATB), **C)** Combination-treated tumor-bearing mice (Combo, Tumor Bearing; CTB). In each case, the directionality of regulation compared to tumor-free controls is designated by arrows.

Appendix Figure S15.

Heat map of DEGs within the Ctnnb1 gene set (mean z score). Tumor-free control (black checkered), GTx-024-treated tumor-free (blue checkered), tumor-bearing control (black), GTx-024-treated tumor-bearing (blue), AR-42-treated tumor-bearing (red) and Combination-treated tumor-bearing (green).

Appendix Tables

<u>Appendix Table S1A-D (related to Figure 1):</u> p-values from statistical comparisons. One way ANOVA followed by Tukey's multiple comparison test.

A. Terminal Body Weight (Figure 1C)

p-values		Tumor Free		Tumor Bearing			
		Veh	GTx-024	Veh (V)	GTx-024 (G)	AR-42 (A)	Combo
Tumor Free	Veh		0.9523	0.0022	0.0006	0.3525	>0.9999
	GTx-024	0.9523		0.0003	<0.0001	0.08	0.957
Tumor Bearing	Veh (V)	0.0022	0.0003		0.997	0.2572	0.0037
	GTx-024 (G)	0.0006	<0.0001	0.997		0.1096	0.0011
	AR-42 (A)	0.3525	0.08	0.2572	0.1096		0.4089
	Combo	>0.9999	0.957	0.0037	0.0011	0.4089	

B. Tumor Volume (Figure 1D)

p-values		Tumor Bearing			
		Veh (V)	GTx-024 (G)	AR-42 (A)	Combo
Tumor Bearing	Veh (V)		0.9167	0.94	0.7914
	GTx-024 (G)	0.9167		0.9998	0.9917
	AR-42 (A)	0.94	0.9998		0.9837
	Combo	0.7914	0.9917	0.9837	

C. Gastroc Mass (Figure 1E)

p-values		Tumor Free		Tumor Bearing			
		Veh	GTx-024	Veh (V)	GTx-024 (G)	AR-42 (A)	Combo
Tumor Free	Veh		0.5108	0.0872	0.0499	0.8541	0.9966
	GTx-024	0.5108		0.0016	0.0008	0.0846	0.8341
Tumor Bearing	Veh (V)	0.0872	0.0016		0.9998	0.6137	0.0424
	GTx-024 (G)	0.0499	0.0008	0.9998		0.4605	0.024
	AR-42 (A)	0.8541	0.0846	0.6137	0.4605		0.6309
	Combo	0.9966	0.8341	0.0424	0.024	0.6309	

D. Quadriceps Mass (Figure 1F)

p-values		Tumor Free		Tumor Bearing			
		Veh	GTx-024	Veh (V)	GTx-024 (G)	AR-42 (A)	Combo
Tumor Free	Veh		0.3151	0.1604	0.0034	0.7106	>0.9999
	GTx-024	0.3151		0.0015	<0.0001	0.0219	0.3545
Tumor Bearing	Veh (V)	0.1604	0.0015		0.5922	0.9042	0.2015
	GTx-024 (G)	0.0034	<0.0001	0.5922		0.1207	0.0056
	AR-42 (A)	0.7106	0.0219	0.9042	0.1207		0.7563
	Combo	>0.9999	0.3545	0.2015	0.0056	0.7563	

<u>Appendix Table S2A-D (related to Figure 2):</u> p-values from statistical comparisons. One way ANOVA followed by Tukey's multiple comparison test.

A. Terminal Body Weight (Figure 2A)

p-values		Tumor Free		Tumor Bearing			
		Veh	GTx-024	Veh (V)	GTx-024 (G)	AR-42 (A)	Combo
Tumor Free	Veh		0.9986	<0.0001	<0.0001	<0.0001	0.0116
	GTx-024	0.9986		<0.0001	<0.0001	<0.0001	0.0033
Tumor Bearing	Veh (V)	<0.0001	<0.0001		0.9998	0.2062	0.0017
	GTx-024 (G)	<0.0001	<0.0001	0.9998		0.0722	0.0002
	AR-42 (A)	<0.0001	<0.0001	0.2062	0.0722		0.3422
	Combo	0.0116	0.0033	0.0017	0.0002	0.3422	

B. Gastroc Mass (Figure 2B)

p-values		Tumor Free		Tumor Bearing			
		Veh	GTx-024	Veh (V)	GTx-024 (G)	AR-42 (A)	Combo
Tumor Free	Veh		0.5864	<0.0001	<0.0001	0.0068	0.0158
	GTx-024	0.5864		<0.0001	<0.0001	<0.0001	<0.0001
Tumor Bearing	Veh (V)	<0.0001	<0.0001		0.9871	0.0933	0.0442
	GTx-024 (G)	<0.0001	<0.0001	0.9871		0.0085	0.0031
	AR-42 (A)	0.0068	<0.0001	0.0933	0.0085		0.9993
	Combo	0.0158	<0.0001	0.0442	0.0031	0.9993	

C. Quadriceps Mass (Figure 2C)

p-values		Tumor Free		Tumor Bearing			
		Veh	GTx-024	Veh (V)	GTx-024 (G)	AR-42 (A)	Combo
Tumor Free	Veh		0.9961	<0.0001	<0.0001	<0.0001	0.0009
	GTx-024	0.9961		<0.0001	<0.0001	<0.0001	0.0002
Tumor Bearing	Veh (V)	<0.0001	<0.0001		0.9977	0.1	0.0045
	GTx-024 (G)	<0.0001	<0.0001	0.9977		0.0166	0.0003
	AR-42 (A)	<0.0001	<0.0001	0.1	0.0166		0.7894
	Combo	0.0009	0.0002	0.0045	0.0003	0.7894	

D. Grip Strength (Figure 2D)

p-values		Tumor Free		Tumor Bearing			
		Veh	GTx-024	Veh (V)	GTx-024 (G)	AR-42 (A)	Combo
Tumor Free	Veh		0.2964	0.177	0.8912	0.9941	0.3545
	GTx-024	0.2964		0.0005	0.0158	0.4868	0.9996
Tumor Bearing	Veh (V)	0.177	0.0005		0.6028	0.0278	0.0003
	GTx-024 (G)	0.8912	0.0158	0.6028		0.4645	0.0133
	AR-42 (A)	0.9941	0.4868	0.0278	0.4645		0.5762
	Combo	0.3545	0.9996	0.0003	0.0133	0.5762	

Appendix Table S3A-D (related to Figure 3): p-values from statistical comparisons. One way ANOVA followed by Tukey's multiple comparison test.

A. Terminal Bodyweight (Figure 3A)

p-values		Tumor Free	Tumor Bearing			
		Veh	Veh (V)	GTx-024 (G)	AR-42 (A)	Combo
Tumor Free	Veh		0.0224	0.653	0.3737	0.9461
Tumor Bearing	Veh (V)	0.0224		0.2304	0.487	0.0002
	GTx-024 (G)	0.653	0.2304		0.9846	0.0977
	AR-42 (A)	0.3737	0.487	0.9846		0.0233
	Combo	0.9461	0.0002	0.0977	0.0233	

B. Gastroc Mass (Figure 3B)

p-values		Tumor Free	Tumor Bearing			
		Veh	Veh (V)	GTx-024 (G)	AR-42 (A)	Combo
Tumor Free	Veh		0.0066	0.6483	0.0772	0.827
Tumor Bearing	Veh (V)	0.0066		0.0802	0.7778	<0.0001
	GTx-024 (G)	0.6483	0.0802		0.5639	0.039
	AR-42 (A)	0.0772	0.7778	0.5639		0.0004
	Combo	0.827	<0.0001	0.039	0.0004	

C. Quadriceps Mass (Figure 3C)

p-values		Tumor Free	Tumor Bearing			
		Veh	Veh (V)	GTx-024 (G)	AR-42 (A)	Combo
Tumor Free	Veh		0.0129	0.3377	0.2743	0.5912
Tumor Bearing	Veh (V)	0.0129		0.388	0.5257	<0.0001
	GTx-024 (G)	0.3377	0.388		0.9997	0.0014
	AR-42 (A)	0.2743	0.5257	0.9997		0.001
	Combo	0.5912	<0.0001	0.0014	0.001	

D. Grip Strength (Figure 3D)

p-values		Tumor Free	Tumor Bearing			
		Veh	Veh (V)	GTx-024 (G)	AR-42 (A)	Combo
Tumor Free	Veh		0.0011	0.0137	0.484	0.991
Tumor Bearing	Veh (V)	0.0011		0.8763	0.0248	<0.0001
	GTx-024 (G)	0.0137	0.8763		0.2391	0.0003
	AR-42 (A)	0.484	0.0248	0.2391		0.0952
	Combo	0.991	<0.0001	0.0003	0.0952	

Appendix Table S4A-D (related to Figure 4): p-values from statistical comparisons. One way ANOVA followed by Tukey's multiple comparison test.

A. Terminal Bodyweight (Figure 4A)

p-values		Tumor Free	Tumor Bearing				
		Veh	Veh (V)	TFM-4AS-1 (T)	AR-42 (A)	TFM/AR-42	DHT/AR-42
Tumor Free	Veh		0.0038	0.0479	0.068	0.7242	>0.9999
Tumor Bearing	Veh (V)	0.0038		0.8694	0.8391	0.0755	0.0004
	TFM-4AS-1 (T)	0.0479	0.8694		>0.9999	0.50858	0.0092
	AR-42 (A)	0.0681	0.8391	>0.9999		0.598	0.0159
	TFM/AR-42	0.7242	0.0755	0.5085	0.598		0.4998
	DHT/AR-42	>0.9999	0.0004	0.0092	0.0159	0.4998	

B. Gastroc Mass (Figure 4B)

p-values		Tumor Free	Tumor Bearing				
		Veh	Veh (V)	TFM-4AS-1 (T)	AR-42 (A)	TFM/AR-42	DHT/AR-42
Tumor Free	Veh		0.0011	0.0031	0.0609	0.2755	0.7767
Tumor Bearing	Veh (V)	0.0011		0.9966	0.6082	0.1752	0.0135
	TFM-4AS-1 (T)	0.0031	0.9966		0.8566	0.3643	0.0387
	AR-42 (A)	0.0609	0.6082	0.8566		0.9634	0.4518
	TFM/AR-42	0.2755	0.1752	0.3643	0.9634		0.9147
	DHT/AR-42	0.7767	0.0135	0.0387	0.4518	0.9147	

C. Quadriceps Mass (Figure 4C)

p-values		Tumor Free	Tumor Bearing				
		Veh	Veh (V)	TFM-4AS-1 (T)	AR-42 (A)	TFM/AR-42	DHT/AR-42
Tumor Free	Veh		<0.0001	<0.0001	0.0015	0.0115	0.0468
Tumor Bearing	Veh (V)	<0.0001		0.8818	0.424	0.1024	0.016
	TFM-4AS-1 (T)	<0.0001	0.8818		0.9582	0.5776	0.1848
	AR-42 (A)	0.0015	0.424	0.9582		0.9699	0.6819
	TFM/AR-42	0.0115	0.1024	0.5776	0.9699		0.9838
	DHT/AR-42	0.0468	0.016	0.1848	0.6819	0.9838	

D. Grip Strength (Figure 4D)

p-values		Tumor Bearing				
		Veh (V)	TFM-4AS-1 (T)	AR-42 (A)	TFM/AR-42	DHT/AR-42
Tumor Bearing	Veh (V)		0.9582	0.9663	0.0347	0.9999
	TFM-4AS-1 (T)	0.9582		0.6617	0.0044	0.9104
	AR-42 (A)	0.9663	0.6617		0.1486	0.9854
	TFM/AR-42	0.0347	0.0044	0.1486		0.0412
	DHT/AR-42	0.9999	0.9104	0.9854	0.0412	

Appendix Table S5A-B (related to Figure 5): p-values from statistical comparisons. One way ANOVA followed by Dunnett's multiple comparison test.

A. Cachexia-associated markers (Figure 5A)

p-values (Atrogin-1)		Tumor Free		Tumor Bearing			
		Veh	GTx-024	Veh (V)	GTx-024 (G)	AR-42 (A)	Combo
Tumor Bearing	Veh (V)	<0.0001	<0.0001		0.4099	0.0003	<0.0001

p-values (MuRF-1)		Tumor Free		Tumor Bearing			
		Veh	GTx-024	Veh (V)	GTx-024 (G)	AR-42 (A)	Combo
Tumor Bearing	Veh (V)	<0.0001	<0.0001		0.4863	<0.0001	<0.0001

p-values (CEPBδ)		Tumor Free		Tumor Bearing			
		Veh	GTx-024	Veh (V)	GTx-024 (G)	AR-42 (A)	Combo
Tumor Bearing	Veh (V)	<0.0001	<0.0001		0.9377	0.0478	0.0002

B. Androgen receptor (Figure 5B)

p-values		Tumor Free		Tumor Bearing			
		Veh	GTx-024	Veh (V)	GTx-024 (G)	AR-42 (A)	Combo
Tumor Bearing	Veh (V)	0.5444	0.9984		0.5631	0.8676	0.3182

Appendix Table S6A-B (related to Figure 6): p-values from statistical comparisons. One way ANOVA followed by Tukey's multiple comparison test.

A. Spleen Mass (Figure 6D)

p-values		Tumor Free		Tumor Bearing			
		Veh	GTx-024	Veh (V)	GTx-024 (G)	AR-42 (A)	Combo
Tumor Free	Veh		>0.9999	0.0003	<0.0001	<0.0001	<0.0001
	GTx-024	>0.9999		0.0006	<0.0001	<0.0001	<0.0001
Tumor Bearing	Veh (V)	0.0003	0.0006		0.972	0.0568	0.0489
	GTx-024 (G)	<0.0001	<0.0001	0.972		0.1801	0.1574
	AR-42 (A)	<0.0001	<0.0001	0.0568	0.1801		>0.9999
	Combo	<0.0001	<0.0001	0.0489	0.1574	>0.9999	

B. C26 Tumor Tissue (Figure 6E)

p-values		Tumor Bearing			
		Veh (V)	GTx-024 (G)	AR-42 (A)	Combo
Tumor Bearing	Veh (V)		0.9806	0.5557	0.0003
	GTx-024 (G)	0.9806		0.7425	0.0004
	AR-42 (A)	0.5557	0.7425		0.0078
	Combo	0.0003	0.0004	0.0078	

Appendix Table S7 (related to Figure 7G): p-values from statistical comparisons. One way ANOVA followed by Tukey's multiple comparison test.

p-values		Tumor Free		
		Veh		
Target:		IL-6	IL-6RA	GP130
Tumor Free	Veh			
	GTx-024 (G)	0.9976	0.9976	0.9976
Tumor Bearing	Veh (V)	0.789	<0.0001	<0.0001
	GTx-024 (G)	0.1912	<0.0001	<0.0001
	AR-42 (A)	>0.9999	0.2624	0.0665
	Combo	>0.9999	0.6716	0.5507

Appendix Table S8 (related to Figure 8E): p-values from statistical comparisons. One way ANOVA followed by Tukey's multiple comparison test.

p-values		Tumor Free Veh				
Target:		WNT5a	FZD1	GSK3B	CTNNB1	CCND1
Tumor Free	Veh					
	GTx-024 (G)	0.9976	0.9976	0.9976	0.9976	0.9976
Tumor Bearing	Veh (V)	<0.0001	0.0046	0.0069	0.0003	<0.0001
	GTx-024 (G)	<0.0001	0.0261	0.0611	0.0046	<0.0001
	AR-42 (A)	0.3528	0.9917	0.8191	0.0453	0.0025
	Combo	0.6638	0.9655	0.9812	0.4696	0.4108

Appendix Table S9 (related to Figure EV1): p-values from statistical comparisons. One way ANOVA followed by Tukey's multiple comparison test.

A. Terminal Body Weight (Figure EV1A)

p-values		SHAM	ORX	
		Veh	Veh (V)	GTx-024 (G)
SHAM	Veh		<0.0001	0.4436
ORX	Veh (V)	<0.0001		<0.0001
	GTx-024 (G)	0.4436	<0.0001	

B. Gastrocnemius (Figure EV1B)

p-values		SHAM	ORX	
		Veh	Veh (V)	GTx-024 (G)
SHAM	Veh		<0.0001	0.0757
ORX	Veh (V)	<0.0001		<0.0001
	GTx-024 (G)	0.0757	<0.0001	

C. Quadriceps (Figure EV1C)

p-values		SHAM	ORX	
		Veh	Veh (V)	GTx-024 (G)
SHAM	Veh		<0.0001	0.9373
ORX	Veh (V)	<0.0001		<0.0001
	GTx-024 (G)	0.9373	<0.0001	

D. Grip Strength (Figure EV1D)

p-values		SHAM	ORX	
		Veh	Veh (V)	GTx-024 (G)
SHAM	Veh		0.9608	<0.0001
ORX	Veh (V)	0.9608		<0.0001
	GTx-024 (G)	<0.0001	<0.0001	

Appendix Table S10 (related to Figure EV2): p-values from statistical comparisons. One way ANOVA followed by Tukey's multiple comparison test.

A. Luteinizing Hormone (Figure EV2A)

p-values		Tumor Free		Tumor Bearing			
		Veh	GTx-024	Veh (V)	GTx-024 (G)	AR-42 (A)	Combo
Tumor Free	Veh		0.1705	>0.9999	0.4212	0.9984	0.3973
	GTx-024	0.1705		0.2411	0.9996	0.396	0.9984
Tumor Bearing	Veh (V)	>0.9999	0.2411		0.5074	0.9996	0.4909
	GTx-024 (G)	0.4212	0.9996	0.5074		0.686	>0.9999
	AR-42 (A)	0.9984	0.396	0.9996	0.686		0.6801
	Combo	0.3973	0.9984	0.4909	>0.9999	0.6801	

B. Spleen Mass (Figure EV2C)

p-values		Tumor Free		Tumor Bearing			
		Veh	GTx-024	Veh (V)	GTx-024 (G)	AR-42 (A)	Combo
Tumor Free	Veh		0.9998	<0.0001	<0.0001	<0.0001	<0.0001
	GTx-024	0.9998		<0.0001	<0.0001	<0.0001	<0.0001
Tumor Bearing	Veh (V)	<0.0001	<0.0001		0.9503	0.3272	0.2985
	GTx-024 (G)	<0.0001	<0.0001	0.9503		0.8273	0.7977
	AR-42 (A)	<0.0001	<0.0001	0.3272	0.8273		>0.9999
	Combo	<0.0001	<0.0001	0.2985	0.7977	>0.9999	

Appendix Table S11 (related to Figure EV3): p-values from statistical comparisons. One way ANOVA followed by Tukey's multiple comparison test.

A. Tumor Volume (Figure EV3A)

p-values (Study 1)		Tumor Bearing			
		Veh (V)	GTx-024 (G)	AR-42 (A)	Combo
Tumor Bearing	Veh (V)		0.9969	0.9984	0.9783
	GTx-024 (G)	0.9969		>0.9999	>0.9999
	AR-42 (A)	0.9984	>0.9999		0.9999
	Combo	0.9783	>0.9999	0.9999	

p-values (Study 2)		Tumor Bearing			
		Veh (V)	GTx-024 (G)	AR-42 (A)	Combo
Tumor Bearing	Veh (V)		0.4489	0.5526	0.1588
	GTx-024 (G)	0.4489		>0.9999	0.9876
	AR-42 (A)	0.5526	>0.9999		0.9744
	Combo	0.1588	0.9876	0.9744	

B. Adipose Mass (Figure EV3B)

p-values		Tumor Free		Tumor Bearing			
		Veh	GTx-024	Veh (V)	GTx-024 (G)	AR-42 (A)	Combo
Tumor Free	Veh		0.8353	<0.0001	<0.0001	<0.0001	<0.0001
	GTx-024	0.8353		<0.0001	<0.0001	<0.0001	<0.0001
Tumor Bearing	Veh (V)	<0.0001	<0.0001		0.7624	0.9804	0.3481
	GTx-024 (G)	<0.0001	<0.0001	0.7624		0.2566	0.0089
	AR-42 (A)	<0.0001	<0.0001	0.9804	0.2566		0.7217
	Combo	<0.0001	<0.0001	0.3481	0.0089	0.7217	

Appendix Table S12 (related to Figure EV4): p-values from statistical comparisons. One way ANOVA followed by Tukey's multiple comparison test.

A. Tumor Mass (Figure EV4C)

p-values		Tumor Bearing			
		Veh (V)	GTx-024 (G)	AR-42 (A)	Combo
Tumor Bearing	Veh (V)		>0.9999	0.8187	>0.9999
	GTx-024 (G)	>0.9999		0.794	>0.9999
	AR-42 (A)	0.8187	0.794		0.8326
	Combo	>0.9999	>0.9999	0.8326	

B. Terminal Bodyweight (Figure EV4D)

p-values		Tumor Free	Tumor Bearing			
		Veh	Veh (V)	GTx-024 (G)	AR-42 (A)	Combo
Tumor	Veh		0.0294	0.0327	0.0015	0.1071
Tumor Bearing	Veh (V)	0.0294		>0.9999	0.7595	0.9766
	GTx-024 (G)	0.0327	>0.9999		0.6801	0.9875
	AR-42 (A)	0.0015	0.7595	0.6801		0.4126
	Combo	0.1071	0.9766	0.9875	0.4126	

C. Gastroc Mass (Figure EV4E)

p-values		Tumor Free	Tumor Bearing			
		Veh	Veh (V)	GTx-024 (G)	AR-42 (A)	Combo
Tumor	Veh		<0.0001	<0.0001	<0.0001	<0.0001
Tumor Bearing	Veh (V)	<0.0001		0.987	0.9894	0.9999
	GTx-024 (G)	<0.0001	0.987		>0.9999	0.9686
	AR-42 (A)	<0.0001	0.9894	>0.9999		0.973
	Combo	<0.0001	0.9999	0.9686	0.973	

Appendix Table S13. Serum Cytokine Panel – Complete Results. Multiplex analysis of diverse serum cytokines[a] at Day 17 sacrifice from Study 2.[b]

	Tumor-free		C-26 Tumor-bearing			
	**Vehicle**	**GTx-024**	**Vehicle**	**GTx-024**	**AR-42**	**Combo**
Eotaxin	715.35 ± 120.63	669.53 ± 100.70	846.40 ± 222.70	876.82 ± 100.53	735.73 ± 110.62	754.99 ± 93.28
G-CSF	248.66 ± 64.60*	338.39 ± 71.70*	12164.11 ± 18944.48	2446.63 ± 1625.70*	2782.18 ± 2191.30	1674.20 ± 1160.74*
GM-CSF	18.71 ± 5.56	13.27 ± 4.62*	21.92 ± 5.36	17.35 ± 4.33	18.70 ± 3.77	20.58 ± 5.40
IFNg	7.19 ± 2.69	5.93 ± 2.14	4.44 ± 2.58	4.12 ± 2.09	4.69 ± 1.11	3.31 ± 1.85
IL-1a	228.75 ± 279.11	60.41 ± 56.12	82.77 ± 61.73	167.80 ± 156.50	143.31 ± 157.59	58.77 ± 40.43
IL-1b	11.26 ± 9.64	14.65 ± 5.66	15.22 ± 8.72	12.12 ± 5.54	20.48 ± 6.25	10.31 ± 6.93
IL-2	18.51 ± 11.07	15.43 ± 6.56	20.81 ± 14.87	15.62 ± 3.41	19.37 ± 6.07	13.74 ± 4.30
IL-3	1.73 ± 0.77	1.10 ± 0.63	10.24 ± 24.68	0.92 ± 0.56	0.72 ± 0.83	0.85 ± 0.44
IL-4	0.98 ± 0.94	0.48 ± 0.37	0.35 ± 0.21	0.23 ± 0.14	0.32 ± 0.19	0.27 ± 0.08
IL-5	7.12 ± 1.97	5.71 ± 3.26	2.18 ± 1.78	1.11 ± 0.66	5.50 ± 4.41	9.23 ± 10.44
IL-6	3.35 ± 1.51*	2.45 ± 1.31*	537.66 ± 417.18	397.54 ± 341.43	256.59 ± 183.1	448.16 ± 294.52
IL-7	14.18 ± 8.61	11.51 ± 11.06	11.01 ± 5.26	11.19 ± 8.17	41.51 ± 99.92	63.39 ± 146.65
IL-9	14.87 ± 10.69	8.02 ± 6.78	10.30 ± 8.93	8.69 ± 9.43	11.28 ± 7.68	12.25 ± 8.35
IL-10	12.22 ± 5.83	2.38 ± 1.13	11.14 ± 12.03	14.15 ± 18.01	15.13 ± 11.16	11.37 ± 8.75
IL12 (p40)	41.84 ± 32.05	22.48 ± 13.42	24.13 ± 19.98	27.61 ± 27.95	31.22 ± 24.43	17.07 ± 20.10
IL-12 (p70)	27.70 ± 19.35	10.83 ± 6.12	17.42 ± 15.14	12.62 ± 7.71	10.16 ± 6.16	9.34 ± 2.66
IL-13	33.94 ± 8.04	29.87 ± 12.81	34.04 ± 9.17	31.63 ± 8.22	34.19 ± 8.29	31.96 ± 8.08
IL-15	103.31 ± 50.35	131.33 ± 70.97	84.16 ± 54.06	97.32 ± 39.18	51.57 ± 19.99	517.81 ± 1308.98
IL-17	3.04 ± 2.26	5.01 ± 1.18*	1.30 ± 0.57	1.75 ± 1.28	1.82 ± 0.85	2.11 ± 1.21
IP-10	162.64 ± 43.04	145.68 ± 48.83*	238.29 ± 124.78	154.76 ± 17.98*	215.35 ± 52.46	227.77 ± 45.23
KC	65.92 ± 2 6.47	90.02 ± 17.69	326.10 ± 215.79	288.89 ± 154.46	363.38 ± 200.65	1094.01 ± 528.53*
LIF	2.03 ± 2.17*	2.50 ± 2.34	24.51 ± 11.26	45.26 ± 21.57*	15.79 ± 5.15	28.08 ± 21.16
LIX	3254.87 ± 3474.12	1316.67 ± 1662.66	4211.17 ± 3120.65	5234.39 ± 4771.34	2515.38 ± 3119.67	1663.11 ± 1732.77
MCP-1	56.10 ± 28.48	54.54 ± 7.47	116.59 ± 58.35	88.07 ± 24.41	91.18 ± 40.23	88.57 ± 18.09
M-CSF	47.72 ± 27.44*	27.23 ± 10.09	23.63 ± 8.29	22.23 ± 9.45	20.21 ± 4.63	21.00 ± 4.19

MIG	100.03 ± 20.20	89.29 ± 41.83	42.36 ± 14.76	39.33 ± 12.88	55.17 ± 10.58	90.75 ± 78.56
MIP-1a	108.48 ± 32.73	75.46 ± 29.41	119.93 ± 59.04	108.03 ± 27.91	63.79 ± 19.14	67.82 ± 37.92
MIP-1b	88.59 ± 10.35	87.69 ± 10.61	89.87 ± 24.41	81.67 ± 11.63	76.41 ± 12.81	83.90 ± 9.08
MIP-2	117.91 ± 32.13	136.75 ± 47.91	139.23 ± 33.21	147.63 ± 59.81	146.34 ± 32.60	105.45 ± 37.15
RANTES	29.72 ± 8.43	20.84 ± 10.38	23.70 ± 9.51	20.67 ± 4.57	19.70 ± 4.31	17.64 ± 6.13
TNFa	12.58 ± 4.75	12.15 ± 4.24	17.86 ± 19.40	12.71 ± 5.12	12.77 ± 4.55	11.01 ± 2.09
VEGF	1.05 ± 0.39	0.87 ± 0.32	1.06 ± 0.27	0.87 ± 0.29	1.04 ± 0.24	0.88 ± 0.22

[a] pg/ml; Data expressed as mean ± SD
[b] Treatments (p.o., qd): GTx-024 (15 mg/kg); AR-42 (10 mg/kg), n=6 for tumor free groups and n=7-10 for tumor bearing groups.
*p<0.05, versus tumor-bearing vehicle-treated controls; One-way ANOVA followed by Dunnett's multiple comparison test.

Eotaxin: chemokine (C-C motif) ligand 11; G-CSF: granulocyte colony-stimulating factor; GM-CSF: granulocyte macrophage colony-stimulating factor; IFNγ: interferon gamma; IL-1a: interleukin-1 alpha; IL-1b: interleukin-1 beta; IL-2: interleukin-2; IL-3: interleukin-3; IL-4: interleukin-4; IL-5: interleukin-5; IL-6: interleukin-6; IL-7: interleukin-7; IL-9: interleukin-9; IL-10: interleukin-10; IL-12 (p40): interleukin-12 subunit p40; IL-12 (p70): interleukin-12 subunit p70; IL-13: interleukin-13; IL-15: interleukin-15; IL-17: interleukin-17; IP-10: interferon gamma-induced protein 10; KC: chemokine (C-X-C motif) ligand 1; LIF: leukemia inhibitory factor; LIX: chemokine (C-X-C motif) ligand 5; MCP: monocyte chemoattractant protein-1; M-CSF: macrophage colony-stimulating factor; MIG: monokine induced by gamma interferon, chemokine (C-X-C motif) ligand 9; MIP-1a: macrophage inflammatory protein-1 alpha; MIP-1b: macrophage inflammatory protein-1 beta; MIP-2: macrophage inflammatory protein-2; RANTES: regulated upon activation, normally T-expressed, and presumably secreted, chemokine (C-C motif) ligand 5; TNFα: tumor necrosis factor-alpha; VEGF: vascular endothelial growth factor.

Appendix Table S14. Primer sequences.

Target	Accession Number	Primer		Amplicon size (bp)	Sequence	Reference
		Forward (5' to 3')	Reverse (5' to 3')			
AR	NM_013476.4	GCCTCCGAAGTGTGGTATCC	CCTGGTACTGTCCAAACGCA	138	2457/2476, 2594/2575	
C/EBPδ	NM_007679.4	CGACTTCAGCGCCTACATTGA	CTAGCGACAGACCCCACAC	171	216/236, 386/368	Primer Bank ID 31560718a1
Fbxo32/ Atrogin1	NM_026346.3	TTCAGCAGCCTGAACTACGA	AGTATCCATGGCGCTCCTTC	139	435/454, 573/554	
Trim63/ MuRF-1	NM_001039048.2	GTGACCAAGGAGAATAGCCAC	ATCAGAGCCTCGATGAAGCC	149	693/713, 841/822	
B-actin	NM_001101.3	CATGTACGTTGCTATCCAGGC	CTCCTTAATGTCACGCACGAT	250	477-497/706-726	Primer Bank ID 4501885a1

Appendix Table S15. Post-alignment quality control metrics for RNA-seq dataset. Adaptor sequences were trimmed from reads with Trimmomatic prior to alignment to mm10, and post-alignment metrics were calculated with samtools. Bed files used to calculate proportions of genic and exonic reads were obtained from the known Gene table associated with the UCSC Genes track (mm10).

Metric	Mean	SD	Min	Max
Total Reads (R1+R2)	44,883,616	8,088,060	34,227,544	67,059,036
Reads After Adaptor Trimming	43,026,750	7,833,050	33,101,092	65,368,280
Duplicate Reads (%)	29.8%	4.3%	23.1%	40.5%
Mapped Reads (%)	89.0%	1.3%	86.5%	91.5%
Genic Reads (%)	84.0%	1.4%	81.2%	86.6%
Exonic Reads (%)	82.0%	1.5%	79.2%	84.9%

Appendix References

Bolger AM, Lohse M, Usadel B (2014) Trimmomatic: a flexible trimmer for Illumina sequence data. Bioinformatics 30: 2114-2120

Bonetto A, Aydogdu T, Kunzevitzky N, Guttridge DC, Khuri S, Koniaris LG, Zimmers TA (2011) STAT3 activation in skeletal muscle links muscle wasting and the acute phase response in cancer cachexia. PloS one 6: e22538

Cheng H, Xie Z, Jones WP, Wei XT, Liu Z, Wang D, Kulp SK, Wang J, Coss CC, Chen CS et al (2016) Preclinical Pharmacokinetics Study of R- and S-Enantiomers of the Histone Deacetylase Inhibitor, AR-42 (NSC 731438), in Rodents. The AAPS journal 18: 737-745

Coss CC, Jones A, Dalton JT (2016) Pharmacokinetic drug interactions of the selective androgen receptor modulator GTx-024(Enobosarm) with itraconazole, rifampin, probenecid, celecoxib and rosuvastatin. Investigational new drugs 34: 458-467

Kim J, Wu D, Hwang DJ, Miller DD, Dalton JT (2005) The para substituent of S-3-(phenoxy)-2-hydroxy-2-methyl-N-(4-nitro-3-trifluoromethyl-phenyl)-propionamid es is a major structural determinant of in vivo disposition and activity of selective androgen receptor modulators. The Journal of pharmacology and experimental therapeutics 315: 230-239

Kolde R (2015) pheatmap: Pretty Heatmaps.

Lawrence M, Huber W, Pages H, Aboyoun P, Carlson M, Gentleman R, Morgan MT, Carey VJ (2013) Software for computing and annotating genomic ranges. PLoS computational biology 9: e1003118

Li H, Handsaker B, Wysoker A, Fennell T, Ruan J, Homer N, Marth G, Abecasis G, Durbin R, Genome Project Data Processing S (2009) The Sequence Alignment/Map format and SAMtools. Bioinformatics 25: 2078-2079

Liao Y, Smyth GK, Shi W (2013) The Subread aligner: fast, accurate and scalable read mapping by seed-and-vote. Nucleic acids research 41: e108

Love MI, Huber W, Anders S (2014) Moderated estimation of fold change and dispersion for RNA-seq data with DESeq2. Genome biology 15: 550

Narayanan R, Ahn S, Cheney MD, Yepuru M, Miller DD, Steiner MS, Dalton JT (2014) Selective androgen receptor modulators (SARMs) negatively regulate triple-negative breast cancer growth and epithelial:mesenchymal stem cell signaling. PloS one 9: e103202

Robinson MD, McCarthy DJ, Smyth GK (2010) edgeR: a Bioconductor package for differential expression analysis of digital gene expression data. Bioinformatics 26: 139-140

Sborov DW, Canella A, Hade EM, Mo X, Khountham S, Wang J, Ni W, Poi M, Coss C, Liu Z et al (2017) A phase 1 trial of the HDAC inhibitor AR-42 in patients with multiple myeloma and T- and B-cell lymphomas. Leukemia & lymphoma 58: 2310-2318

Schmidt A, Kimmel DB, Bai C, Scafonas A, Rutledge S, Vogel RL, McElwee-Witmer S, Chen F, Nantermet PV, Kasparcova V et al (2010) Discovery of the selective androgen receptor

modulator MK-0773 using a rational development strategy based on differential transcriptional requirements for androgenic anabolism versus reproductive physiology. The Journal of biological chemistry 285**:** 17054-17064

Subramanian A, Tamayo P, Mootha VK, Mukherjee S, Ebert BL, Gillette MA, Paulovich A, Pomeroy SL, Golub TR, Lander ES et al (2005) Gene set enrichment analysis: a knowledge-based approach for interpreting genome-wide expression profiles. Proceedings of the National Academy of Sciences of the United States of America 102**:** 15545-15550

Tseng YC, Kulp SK, Lai IL, Hsu EC, He WA, Frankhouser DE, Yan PS, Mo X, Bloomston M, Lesinski GB et al (2015) Preclinical Investigation of the Novel Histone Deacetylase Inhibitor AR-42 in the Treatment of Cancer-Induced Cachexia. Journal of the National Cancer Institute 107**:** djv274

Appendix Figure S1

A

Dose (mg/kg)	Tissue	C_{max} (nM)	AUC_{all} (nM*h)	C_{avg} (nM)
10	Plasma	1,501.6	10,931.6	455.5
10	Muscle	1,109.5	11,076.7	461.5
20	Plasma	3,858.9	34,369.3	1,432.1
20	Muscle	3,662.4	36,504.2	1,521.0
50	Plasma	7,925.1	74,307.3	3,096.1
50	Muscle	6,367.0	72,624.7	3,026.2

B

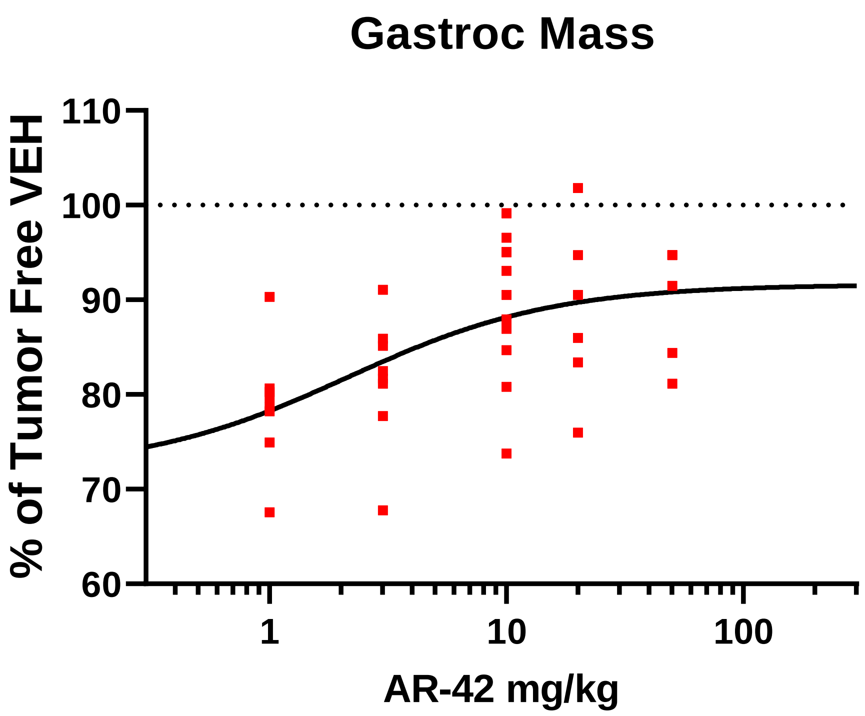

Appendix Figure S2

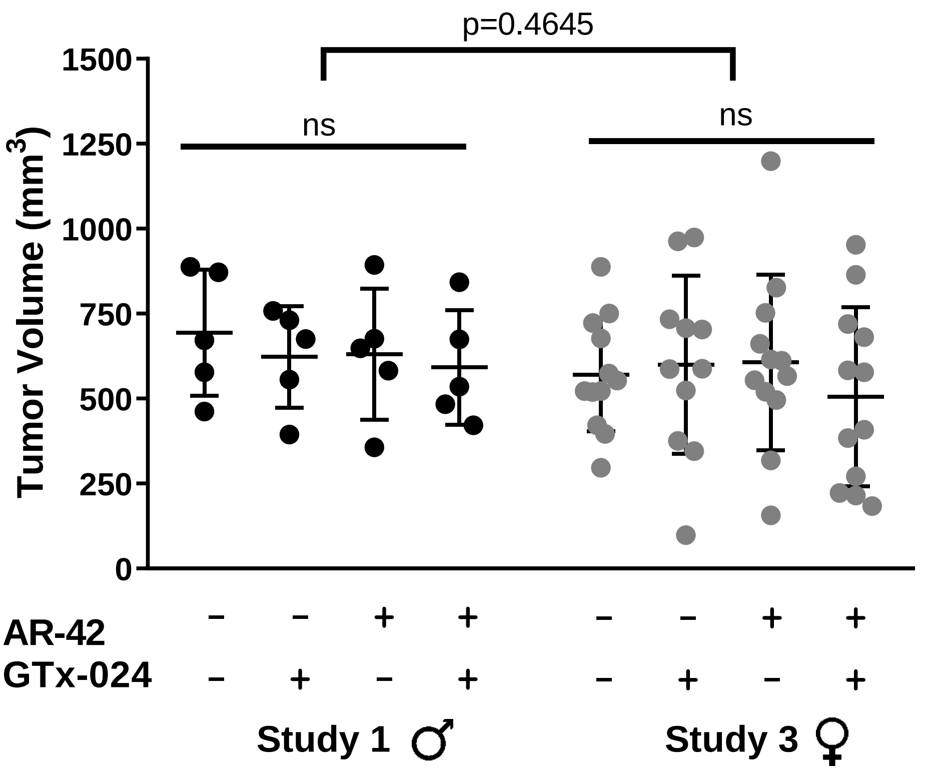

p-values (Study 1)		Tumor Bearing			
		Veh (V)	GTx-024 (G)	AR-42 (A)	Combo
Tumor Bearing	Veh (V)		0.9969	0.9984	0.9783
	GTx-024 (G)	0.9969		>0.9999	>0.9999
	AR-42 (A)	0.9984	>0.9999		0.9999
	Combo	0.9783	>0.9999	0.9999	

p-values (Study 3)		Tumor Bearing			
		Veh (V)	GTx-024 (G)	AR-42 (A)	Combo
Tumor Bearing	Veh (V)		0.989	0.979	0.8926
	GTx-024 (G)	0.989		0.9999	0.7441
	AR-42 (A)	0.979	0.9999		0.6882
	Combo	0.8926	0.7441	0.6882	

Appendix Figure S3

A

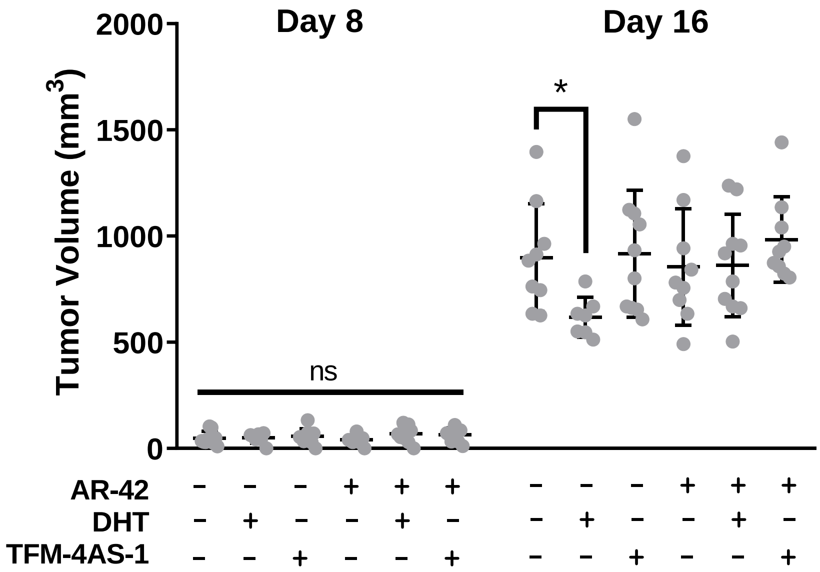

p-values (Day 8)		Tumor Bearing Day 8	Tumor Bearing Day 16
Tumor Bearing	Veh		
	DHT	>0.9999	0.0086
	TFM-4AS-1	>0.9999	0.9998
	AR-42	>0.9999	0.9885
	AR-42/DHT	0.9994	0.9942
	AR-42/TFM-4AS-1	0.9999	0.8315

B

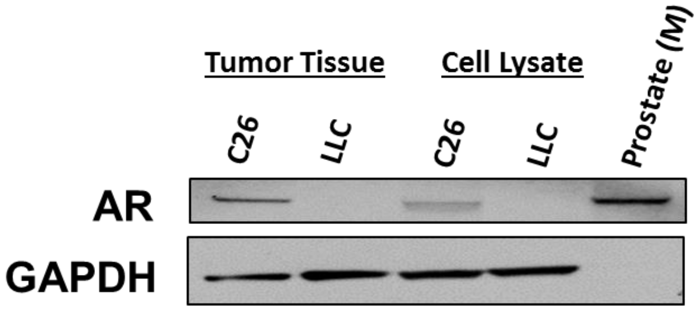

C

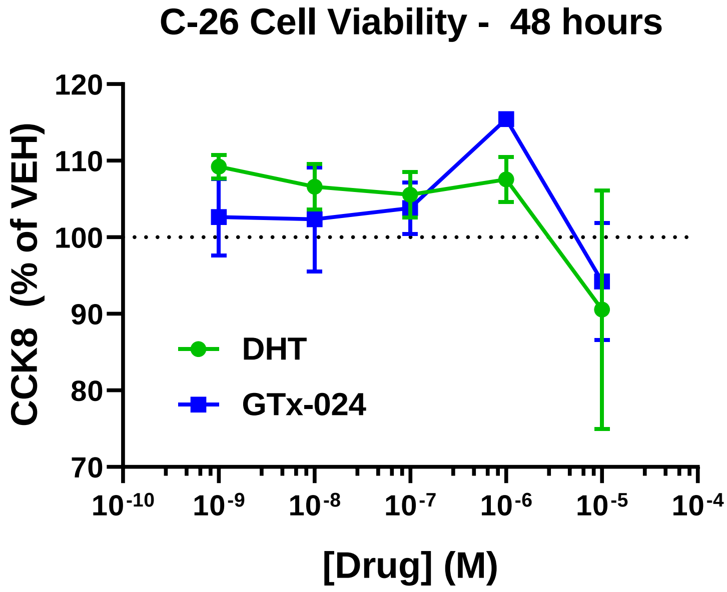

Appendix Figure S4

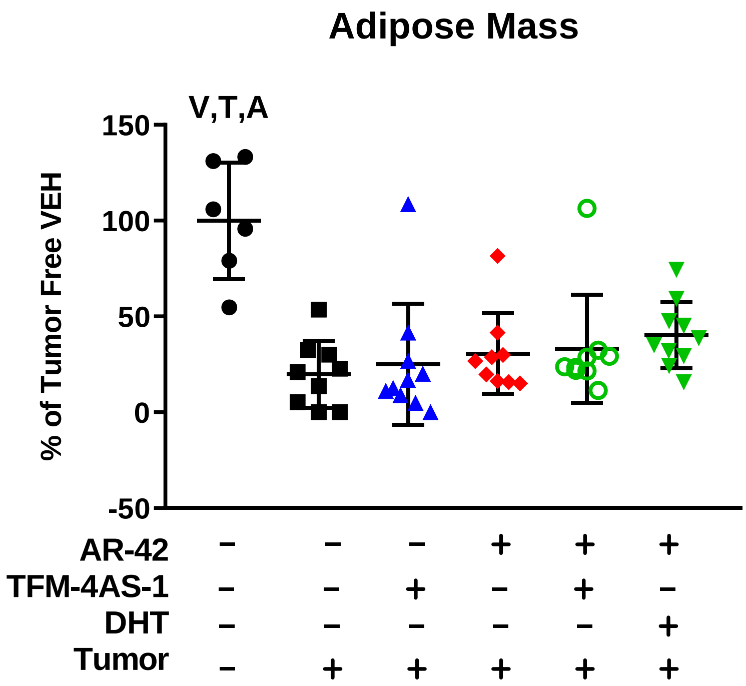

p-values		Tumor Free	Tumor Bearing				
		Veh	Veh	TFM-4AS-1	AR-42	AR-42/ DHT	AR-42/ TFM-4AS-1
Tumor	Veh		<0.0001	<0.0001	<0.0001	0.0003	<0.0001
Tumor Bearing	Veh	<0.0001		0.9971	0.9382	0.4796	0.8578
	TFM-4AS-1	<0.0001	0.9971		0.9966	0.7481	0.9794
	AR-42	<0.0001	0.9382	0.9966		0.9573	>0.9999
	AR-42/DHT	0.0003	0.4796	0.7481	0.9573		0.9895
	AR-42/TFM-4AS-1	<0.0001	0.8578	0.9794	>0.9999	0.9895	

Appendix Figure S5

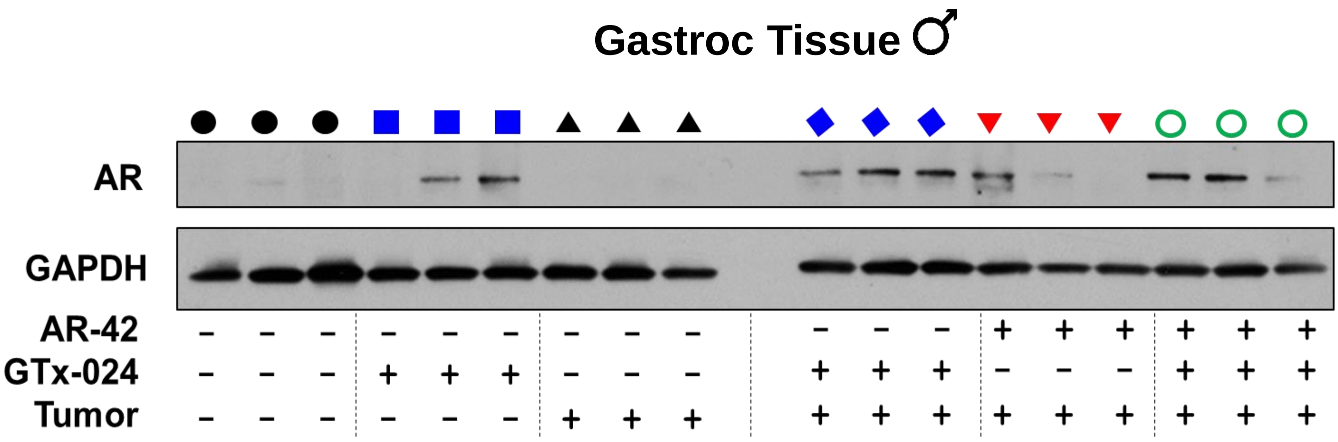

Appendix Figure S6

A

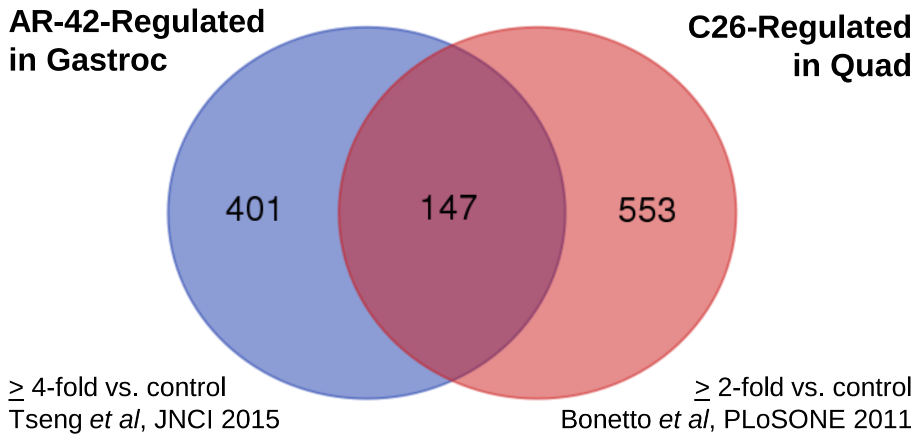

B

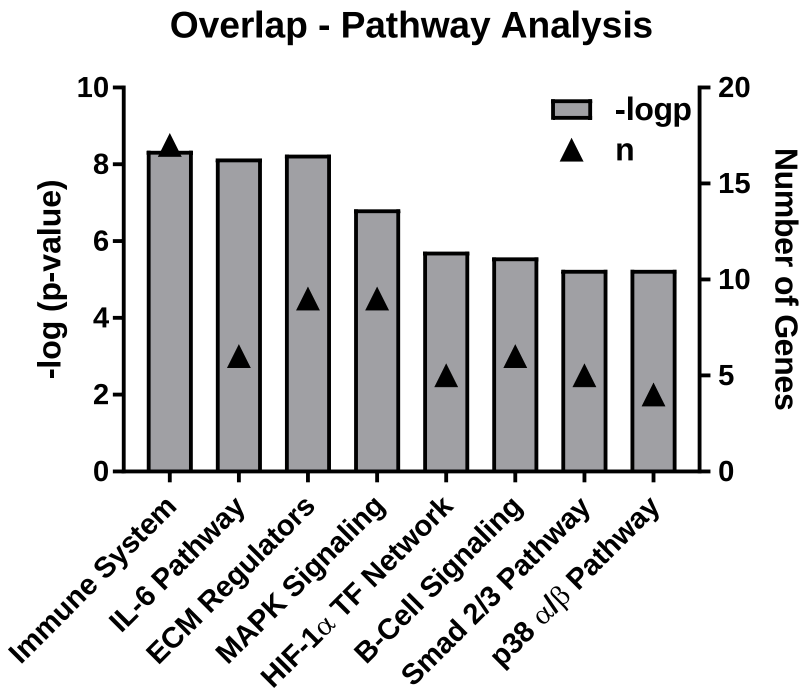

Appendix Figure S7

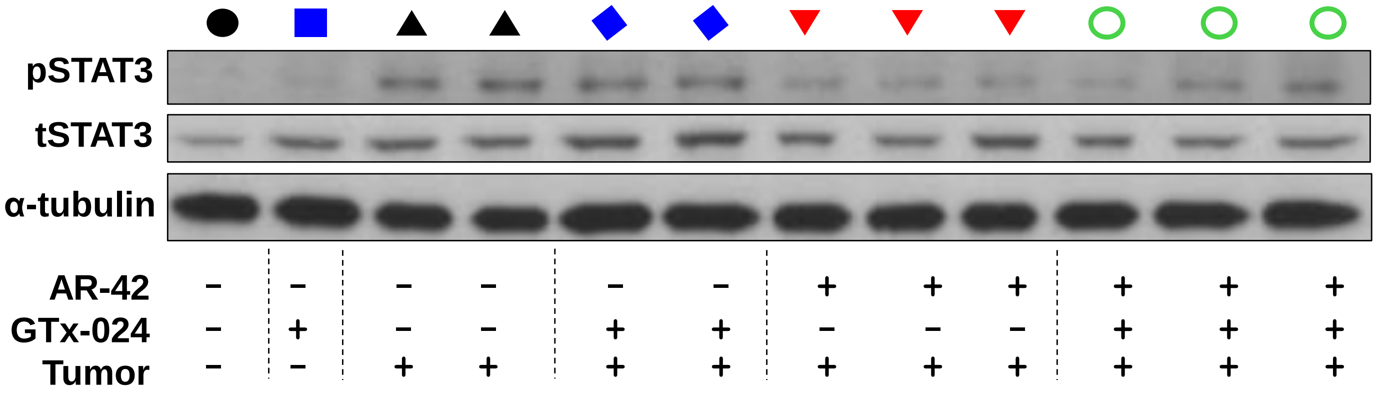

Appendix Figure S8

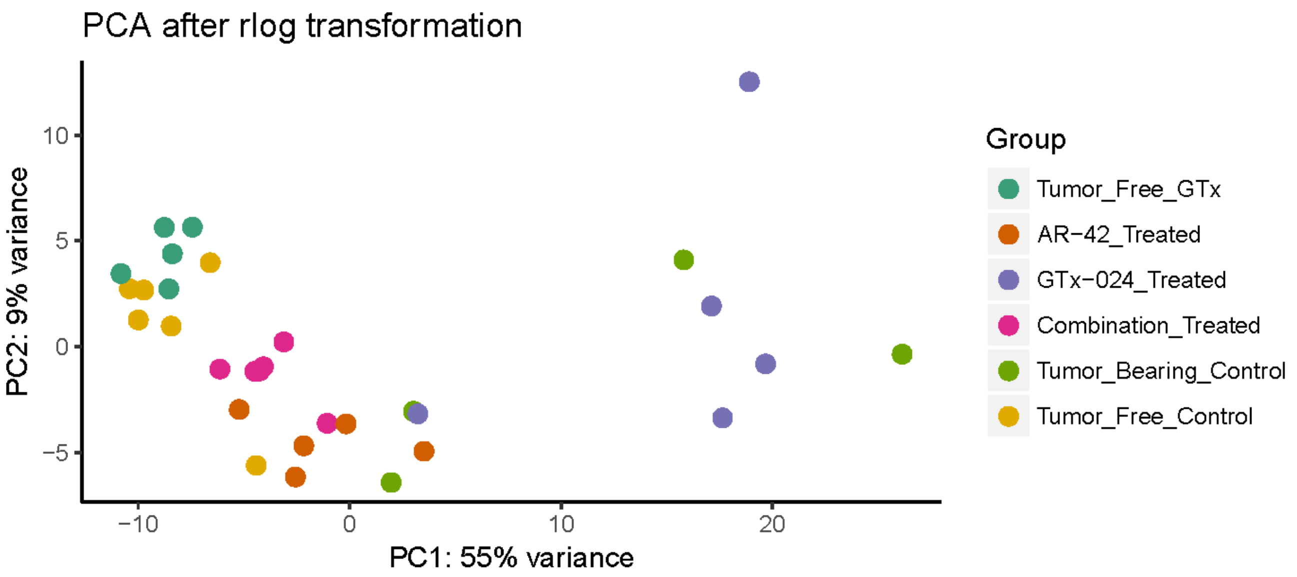

Appendix Figure S9

A

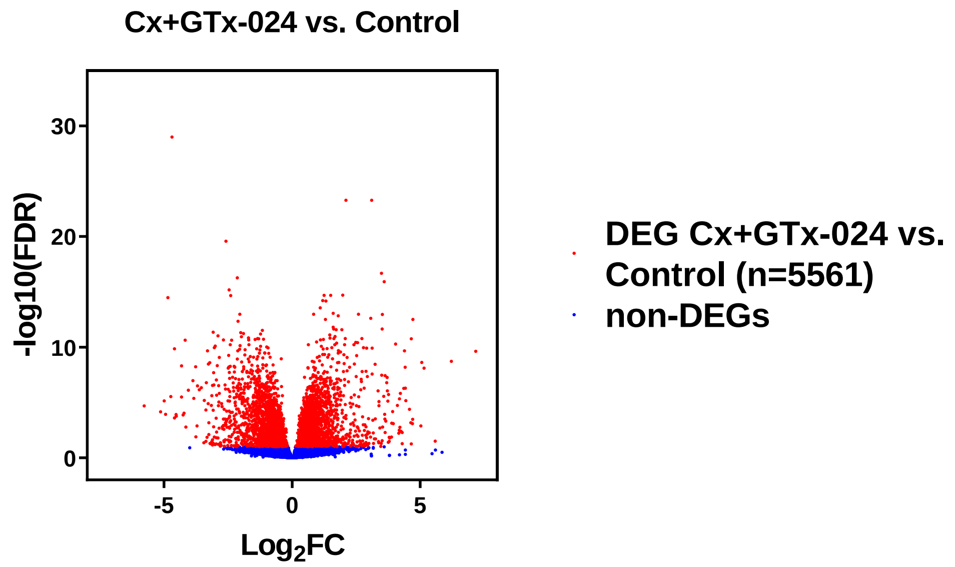

B

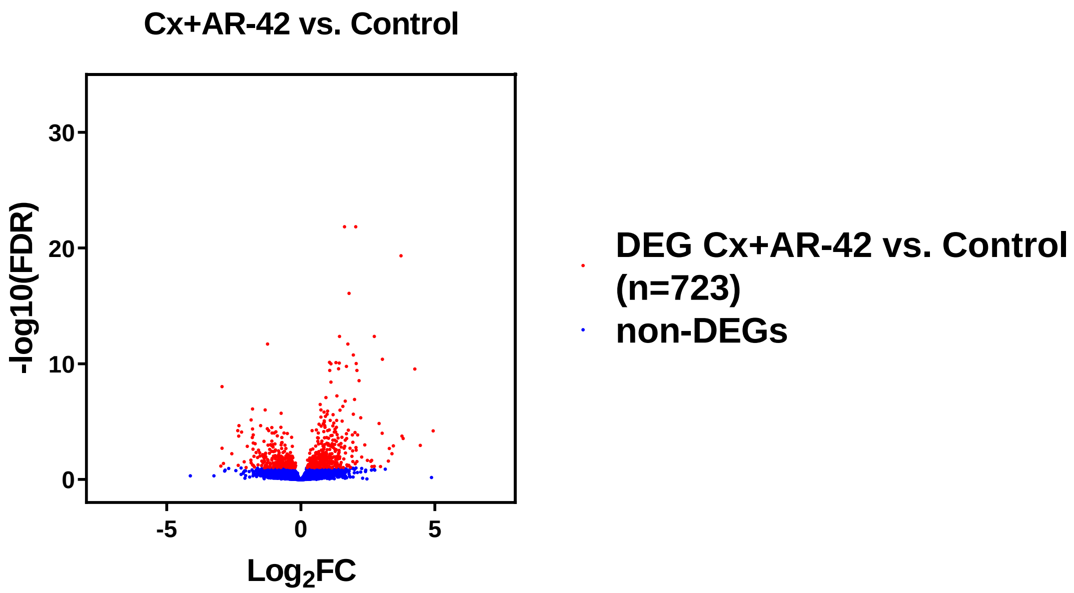

Appendix Figure S10

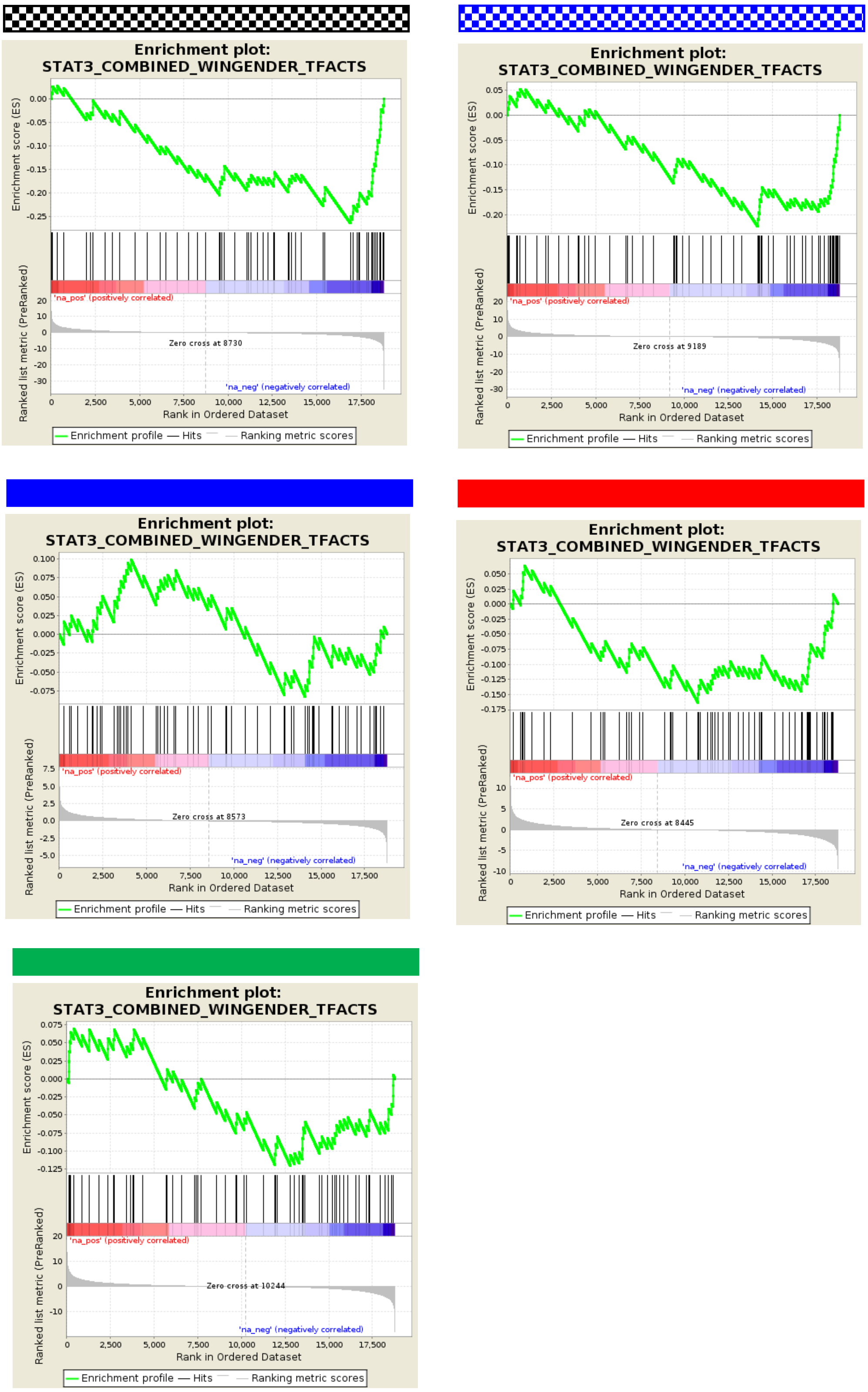

Appendix Figure S11

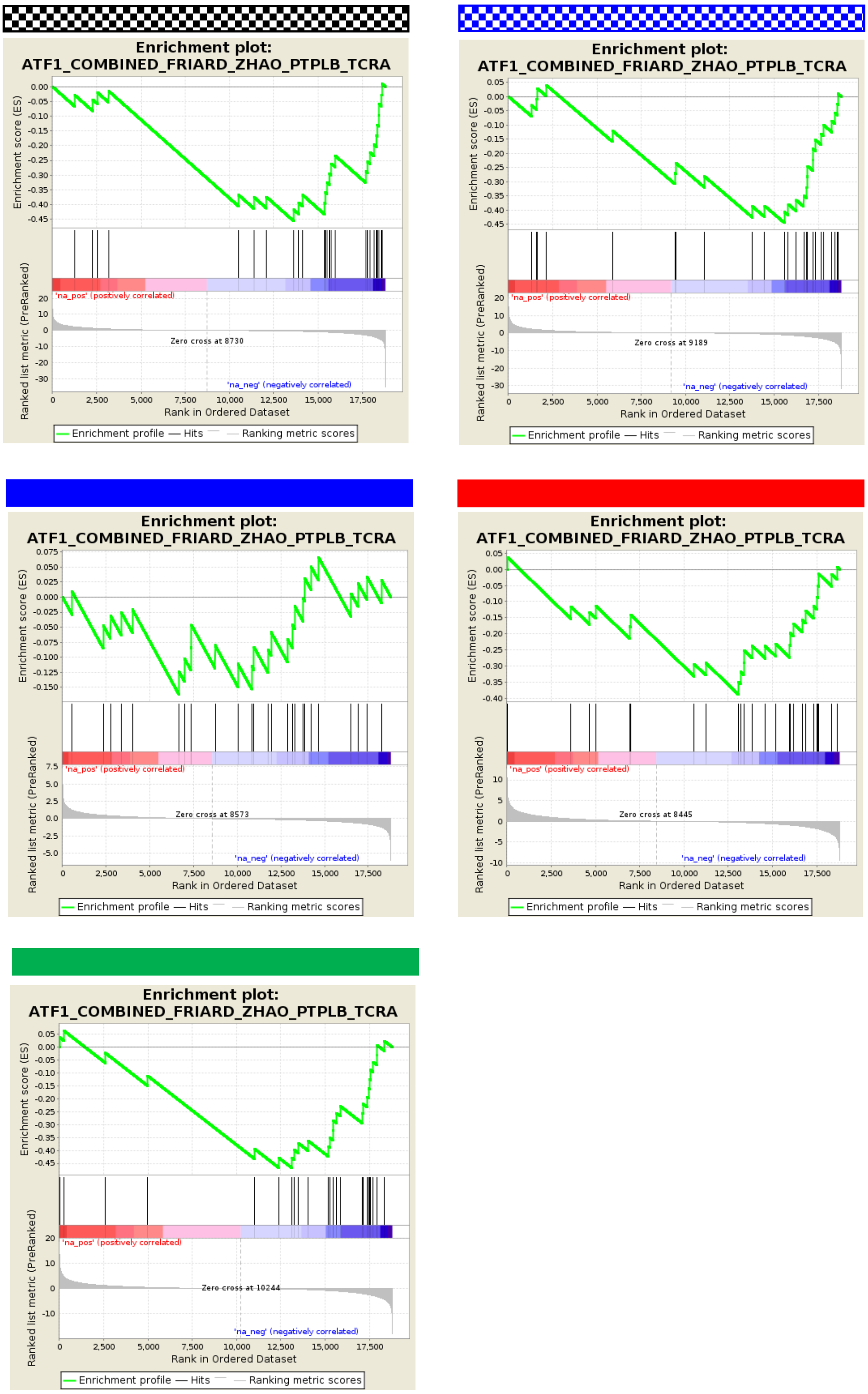

Appendix Figure S12

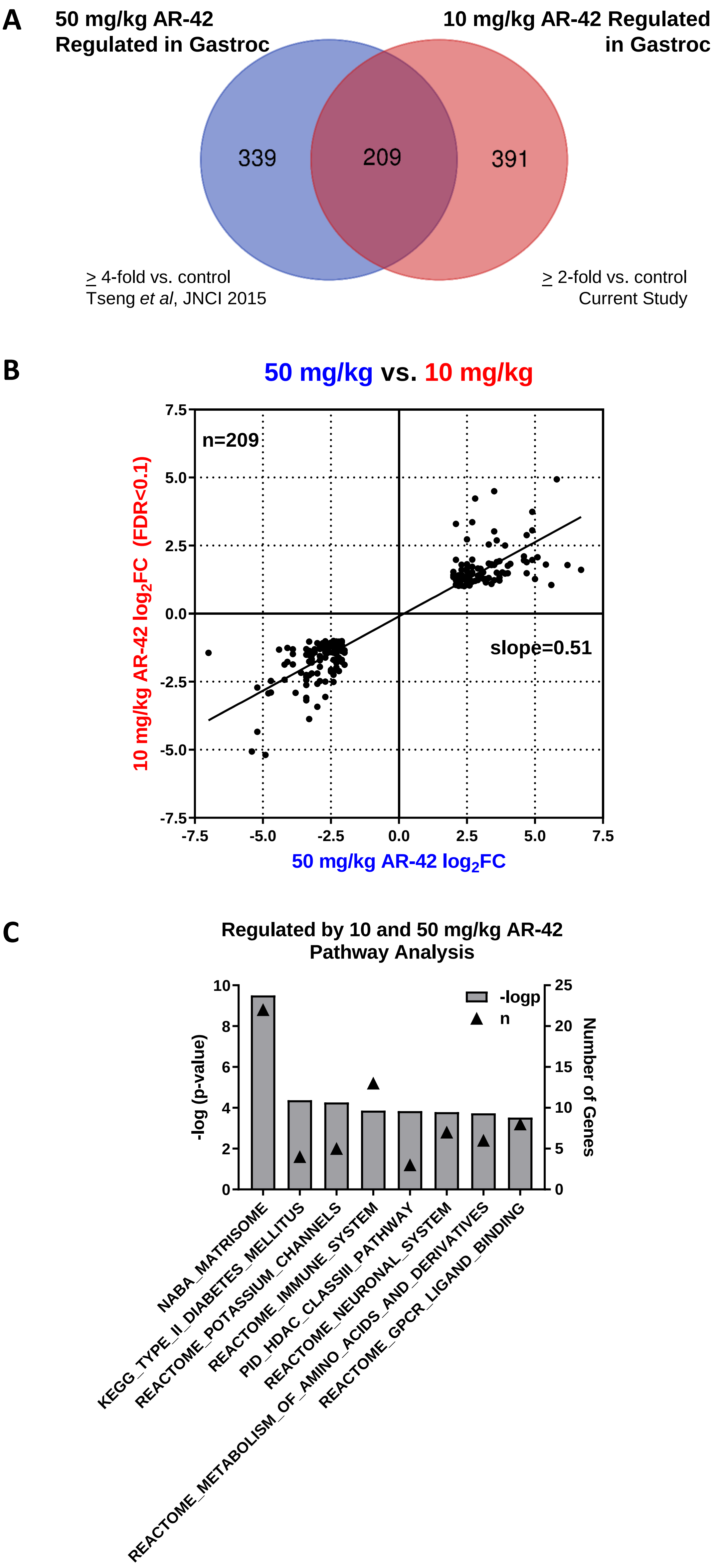

Appendix Figure S13

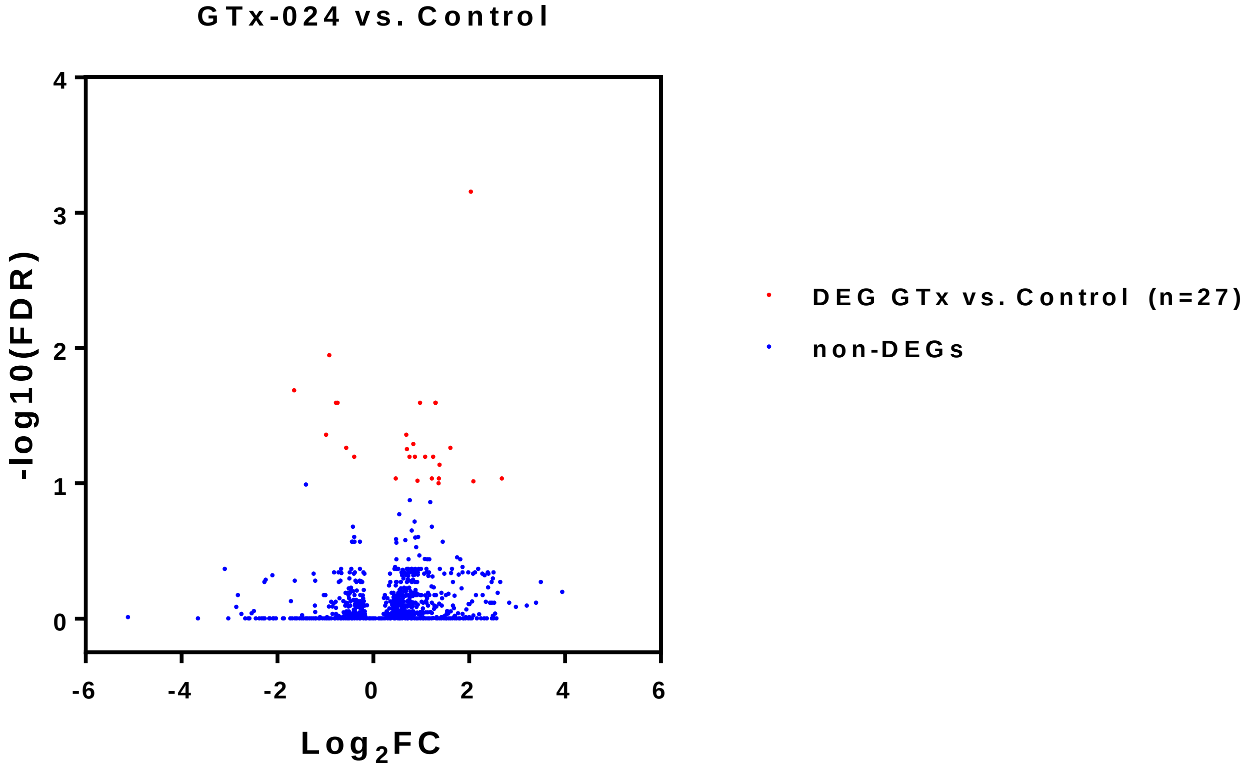

Appendix Figure S14

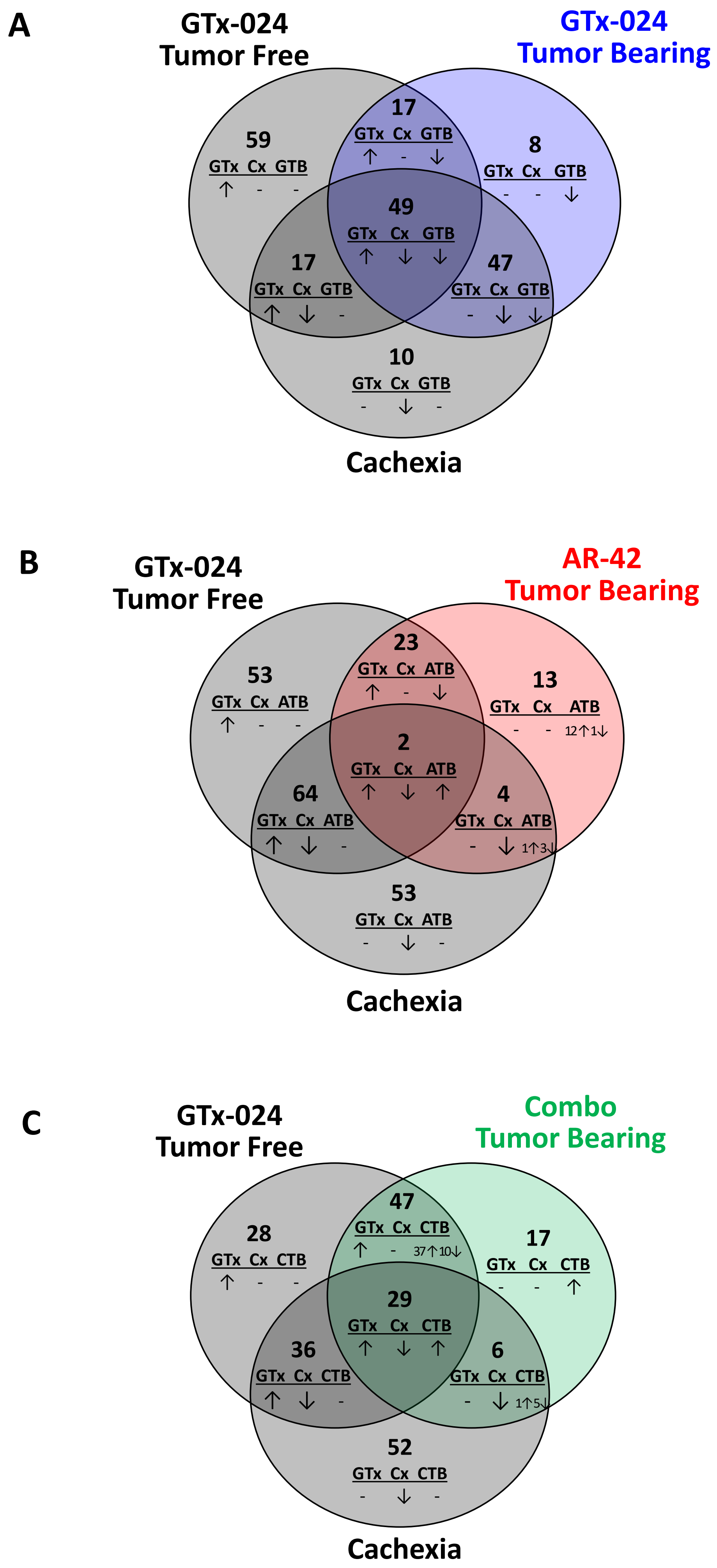

Appendix Figure S15

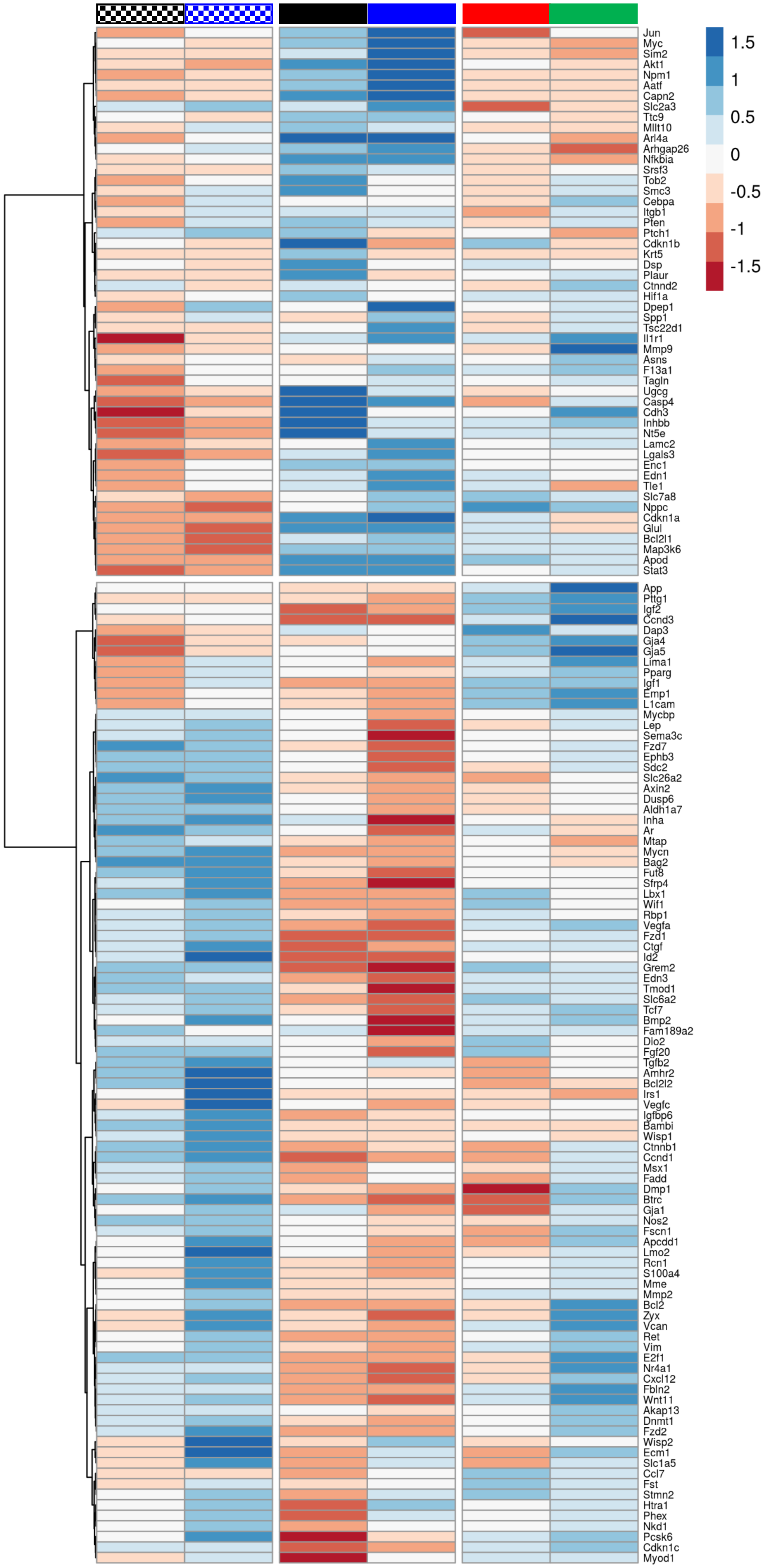

Supplement: Supplementary file 1 — Appendix [file EMMM-12-e9910-s001.pdf]
