# Supplementary material for: Overcoming resistance to anabolic SARM therapy in experimental cancer cachexia with an HDAC inhibitor
Source: EMBO Mol Med. 2020 Jan 13;12(2):e9910. doi: 10.15252/emmm.201809910 (PMC7005646; doi:10.15252/emmm.201809910)
Supplement: Supplementary file 2 — Expanded View Figures PDF [file EMMM-12-e9910-s002.pdf]

Expanded View Figures

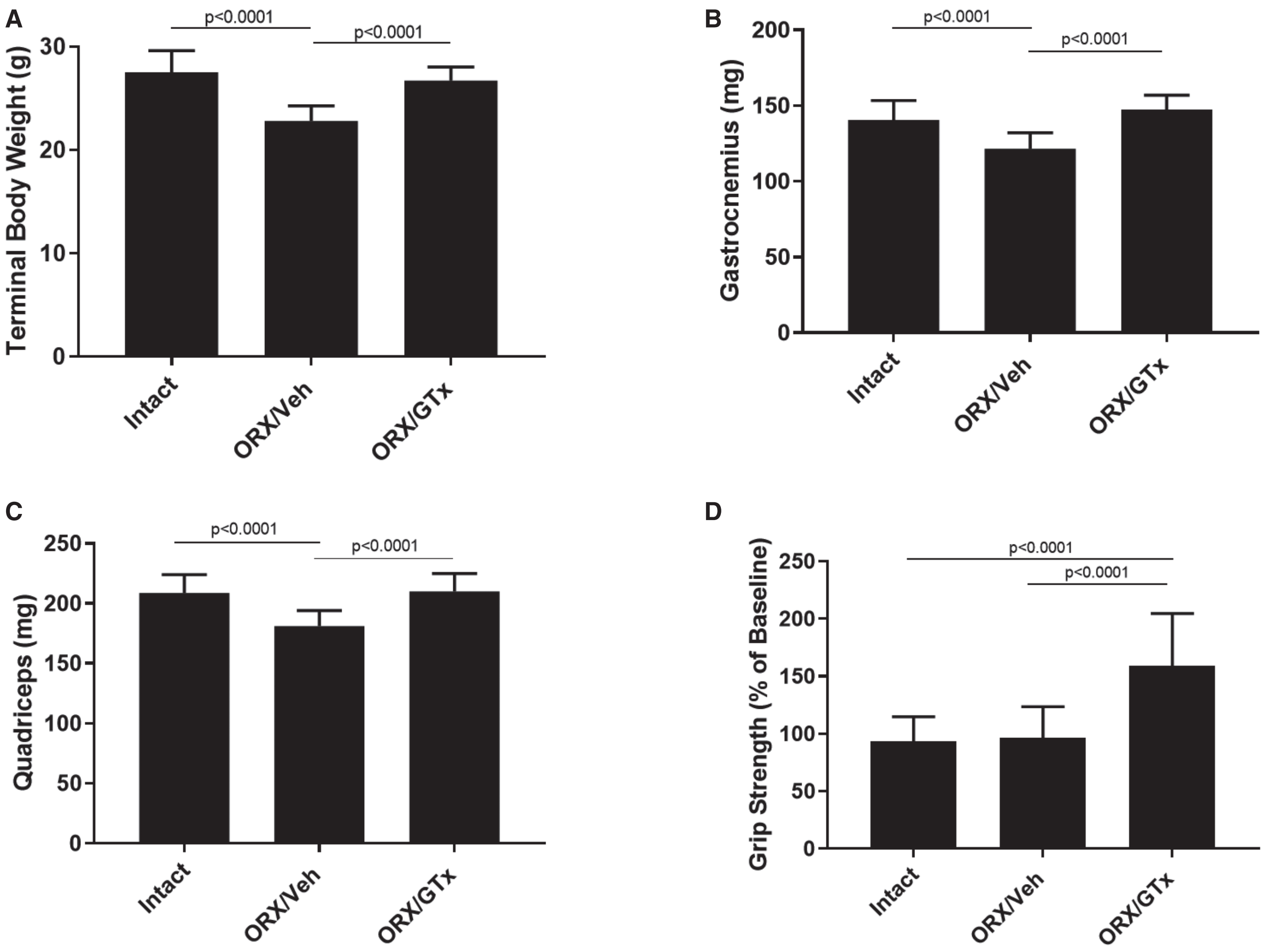

Figure EV1. Confirmation of the anabolic activity of GTx-024.

Orchiectomized (ORX) male CD2F1 mice were treated with GTx-024 (GTx; 15 mg/kg; $n = 12$) or vehicle (Veh; $n = 13$) once daily by oral gavage for 28 days. Sham-orchiectomized (intact) mice ($n = 14$) were treated identically with vehicle.

A Terminal body weights.
B Terminal gastrocnemius weights.
C Terminal quadriceps weights.
D Forelimb grip strength expressed as % baseline defined as (end of study grip strength) × 100/(pre-treatment grip strength).

Data information: *P*-values are provided in Appendix Table S9. One-way ANOVA followed by Tukey's multiple comparisons procedure. Data are presented as means ± SD.

© 2020 The Authors

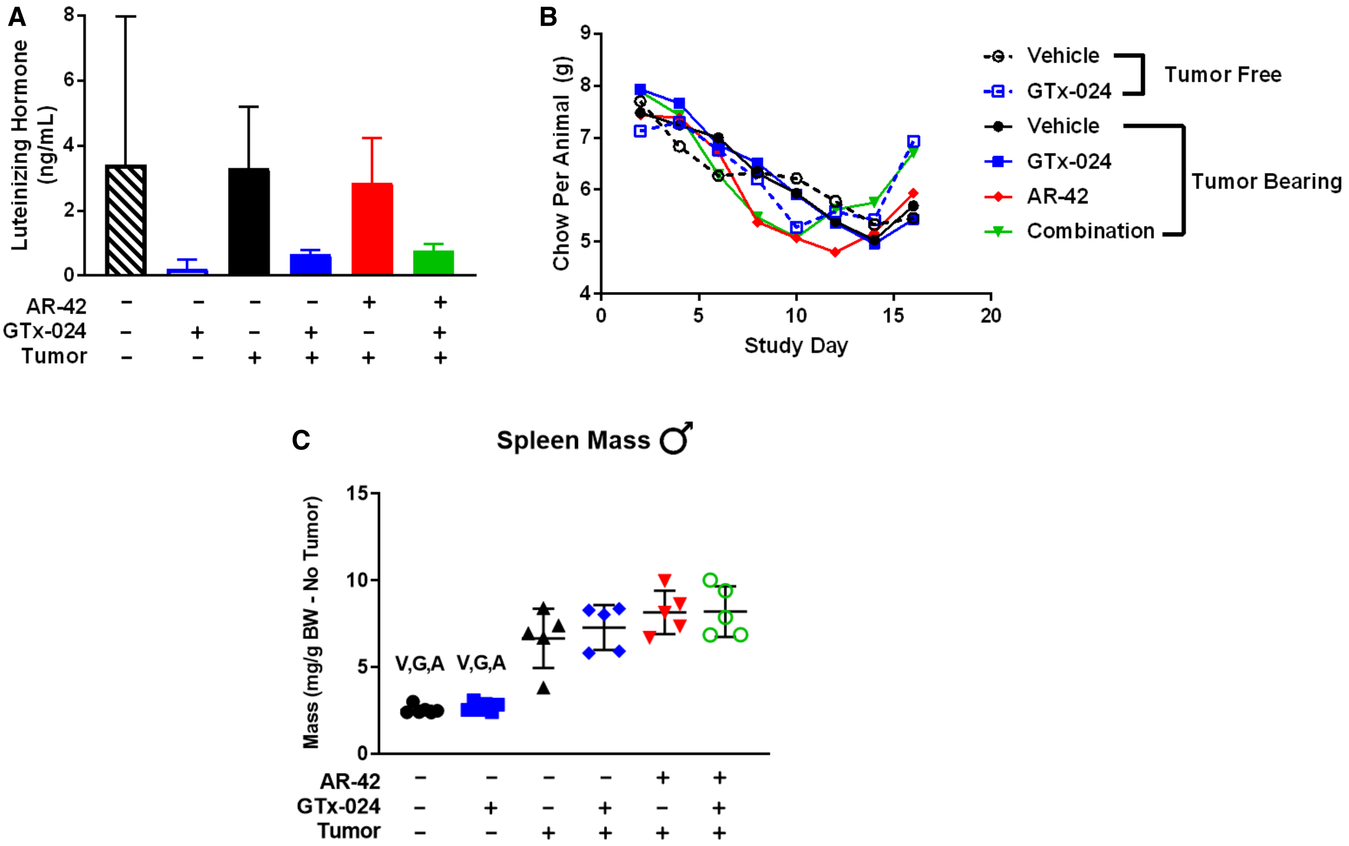

Figure EV2. Anti-cachectic effects of combination therapy in male C-26 tumor-bearing mice.

Study 1, tumor-bearing male mice receiving GTx-024 (15 mg/kg; n = 5), AR-42 (10 mg/kg; n = 5), combination (15 mg/kg GTx-024 and 10 mg/kg AR-42; n = 5), or vehicle (n = 5) and tumor-free mice receiving GTx-024 (15 mg/kg; n = 6) or vehicle (n = 6) were treated daily by oral gavage for 13 days starting 6 days post-injection of C-26 cells.

A Serum-luteinizing hormone levels measured on day 18.
B Per animal food consumption measured every 2 days.
C Spleen weights normalized to tumor mass-corrected terminal body weight.

Data information: V, G, A indicate statically significant differences versus tumor-bearing vehicle-treated, tumor-bearing GTx-024-treated, and tumor-bearing AR-42-treated groups, respectively. P-values are provided in Appendix Table S10, one-way ANOVA followed by Tukey's multiple comparison test. Means ± SD, except for (B) in which error bars are not shown to improve clarity of presentation. BW, body weight.

© 2020 The Authors

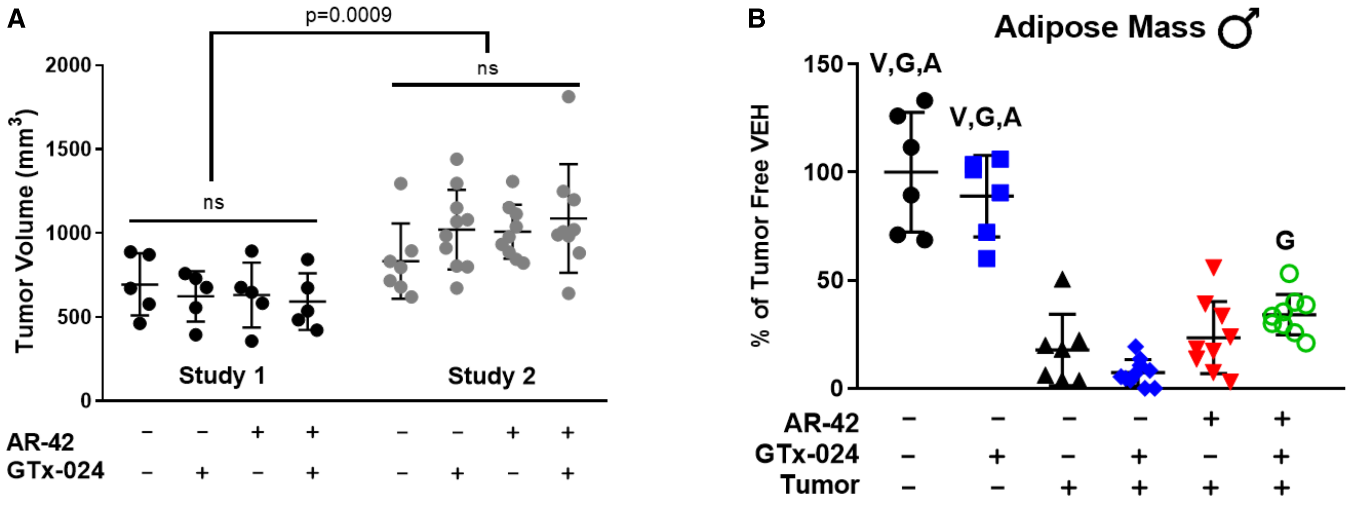

Figure EV3. Terminal tumor volume and fat mass in Study 2.

Study 2, tumor-bearing male mice receiving GTx-024 (15 mg/kg; $n = 10$), AR-42 (10 mg/kg; $n = 9$), combination (15 mg/kg GTx-024 and 10 mg/kg AR-42; $n = 9$) or vehicle ($n = 7$) and tumor-free male mice receiving GTx-024 (15 mg/kg; $n = 6$) or vehicle ($n = 6$) were treated daily by oral gavage for 12 days starting 6 days post-injection of C-26 cells.

A Terminal tumor volume comparisons between the initial (Study 1, day 18) and confirmatory (Study 2, day 17) combination studies. *Statistics:* $P = 0.0009$, Student's *t*-test of combined tumor volumes (Study 1 versus Study 2). ns, no significant differences among treatment groups within each study, one-way ANOVA followed by Tukey's multiple comparison test.

B Terminal epididymal fat pad mass.

Data information: V, G, A indicate statistically significant differences versus tumor-bearing vehicle-treated, tumor-bearing GTx-024-treated, and tumor-bearing AR-42-treated groups, respectively. *P*-values are provided in Appendix Table S11, one-way ANOVA followed by Tukey's multiple comparison test. Data are presented as means ± SD.

 © 2020 The Authors

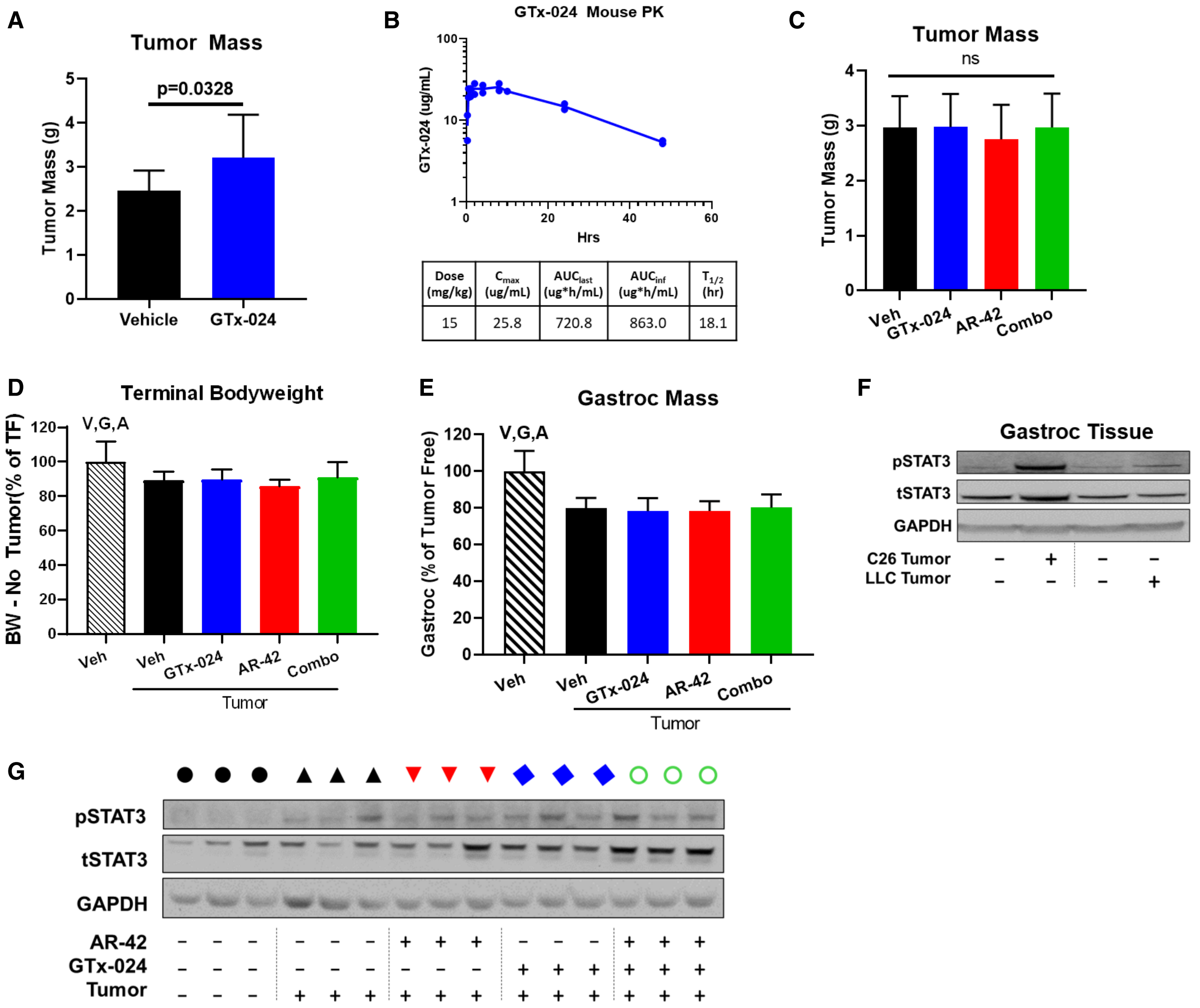

Figure EV4. Anabolic resistance in the LLC model.

Study 5, tumor-bearing male mice receiving GTx-024 (15 mg/kg; n = 12), AR-42 (10 mg/kg; n = 12), combination (15 mg/kg GTx-024 and 10 mg/kg AR-42; n = 12), or vehicle (n = 12) and tumor-free mice receiving vehicle (n = 6) were treated daily by oral gavage for 14 days starting 6 days post-injection of LLC cells.

- A LLC tumor-bearing mice receiving 15 mg/kg GTx-024 monotherapy exhibited larger terminal tumor mass than tumor-bearing vehicle-treated controls.
- B Plasma pharmacokinetics of GTx-024 following a single 15 mg/kg oral dose.
- C–E Repeat of Study 5 with lower dose of GTx-024. Tumor-bearing male mice receiving GTx-024 (0.5 mg/kg; n = 12), AR-42 (10 mg/kg; n = 12), combination (0.5 mg/kg GTx-024 and 10 mg/kg AR-42; n = 12), or vehicle (n = 12) and tumor-free mice receiving vehicle (n = 6) were treated daily by oral gavage for 13 days starting 6 days post-injection of LLC cells. (C) Terminal tumor masses (mean ± SD). ns, no significant differences among treatment groups, one-way ANOVA followed by Tukey's multiple comparison test. (D) Terminal body weights corrected for tumor mass compared to tumor-free controls (mean ± SD). (E) Terminal gastrocnemius mass (mean±SD) compared to tumor-free controls.
- F Western blot of pSTAT3 (Y705) and total (t)STAT3 in pooled gastrocnemius muscle from C-26 and LLC tumor-bearing animals and associated tumor-free controls.
- G Western blot of pSTAT3/tSTAT3 levels in gastrocnemius tissue from representative individual animals from Study 5. Black circle—tumor-free, blue square—tumor-free/GTx-024, black triangle—tumor-bearing, blue diamond—tumor/GTx-024, red triangle—tumor/AR-42, green circle—tumor/combo.

Data information: (A) P = 0.0328 versus tumor-bearing vehicle-treated controls. One-way ANOVA followed by Dunnett's multiple comparison test. (C, D) V, G, A indicate statistically significant differences for tumor-free versus tumor-bearing vehicle-treated (P = 0.0294), tumor-bearing GTx-024-treated (P = 0.0327), and tumor-bearing AR-42-treated groups (P = 0.0015), respectively. P-values are provided in Appendix Table S12. One-way ANOVA followed by Tukey's multiple comparison test. (E) V, G, A indicate statistically significant differences for tumor-free versus tumor-bearing vehicle-treated (P < 0.0001), tumor-bearing GTx-024-treated (P < 0.0001), and tumor-bearing AR-42-treated (P < 0.0001) groups, respectively. P-values are provided in Appendix Table S12. One-way ANOVA followed by Tukey's multiple comparison test.

Source data are available online for this figure.

© 2020 The Authors
