# Supplementary figures and images for: Overcoming resistance to anabolic SARM therapy in experimental cancer cachexia with an HDAC inhibitor
Source: EMBO Mol Med. 2020 Jan 13;12(2):e9910. doi: 10.15252/emmm.201809910 (PMC7005646; doi:10.15252/emmm.201809910)

Figure EV4F

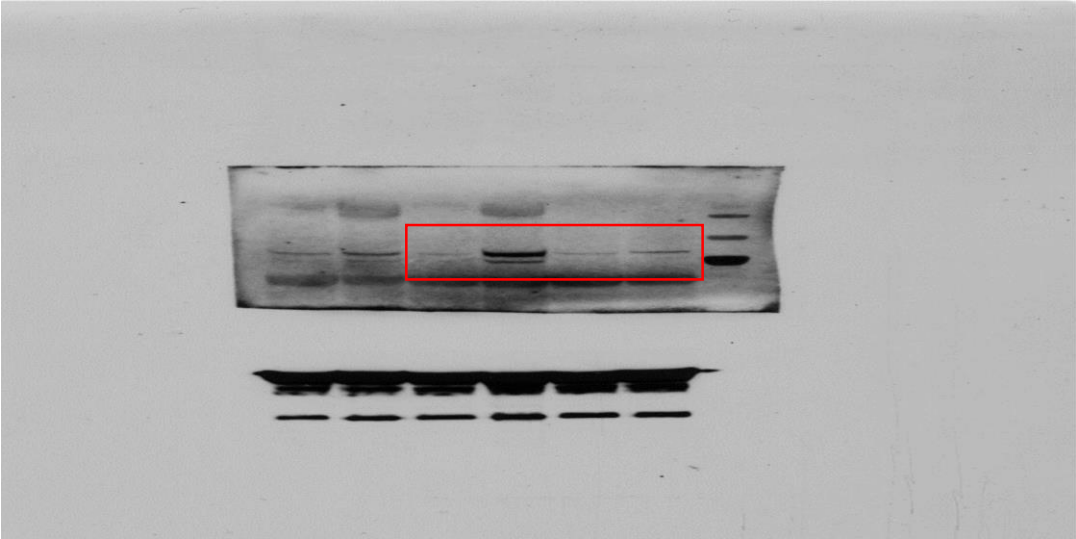

pSTAT3

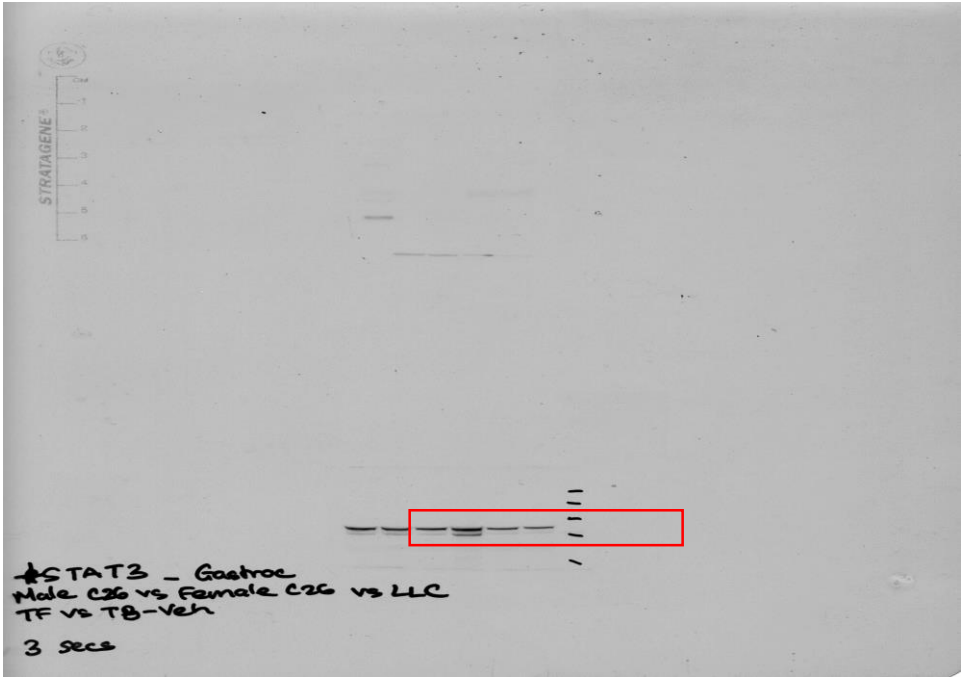

STAT3

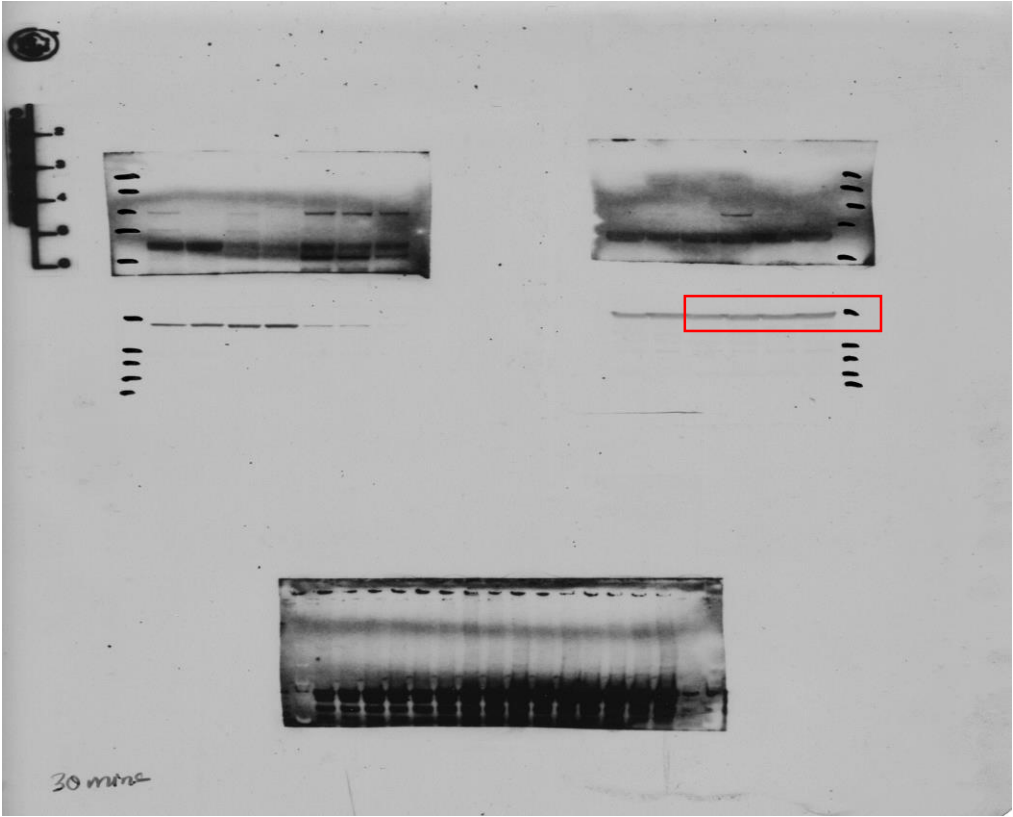

GAPDH

Supplement: Supplementary file 3 — Source Data for Expanded View and Appendix [file EMMM-12-e9910-s004.zip › EV-Appendix-SD/Source_Data_EV4F.pdf]

Figure EV4G

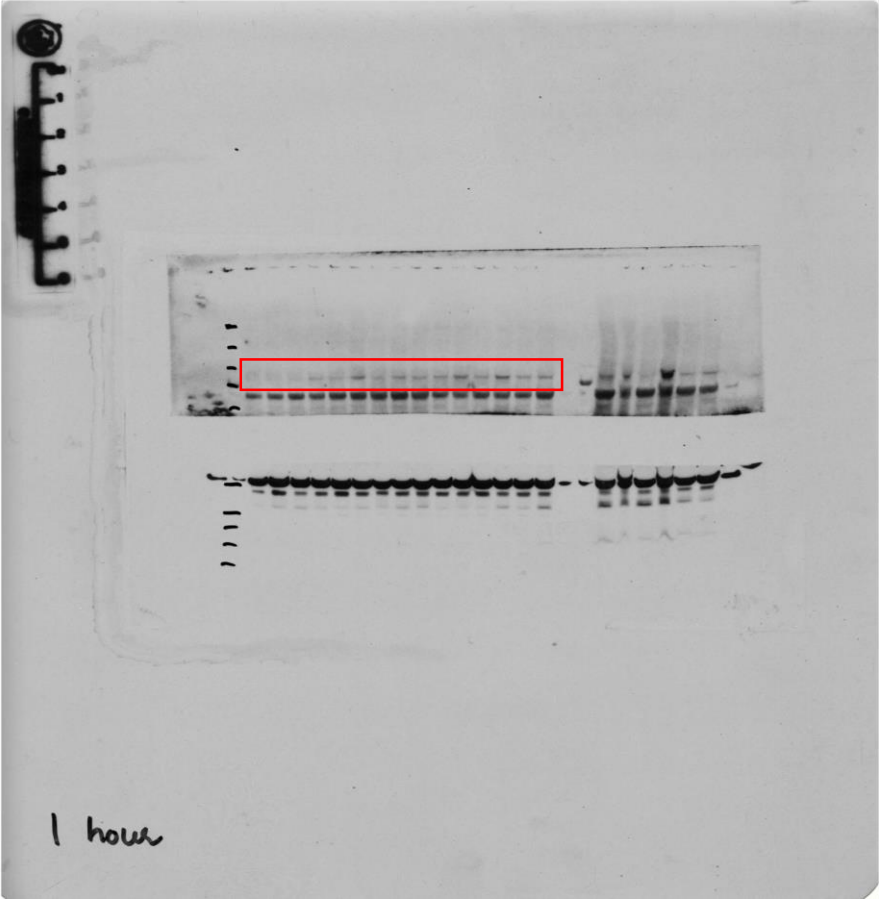

pSTAT3

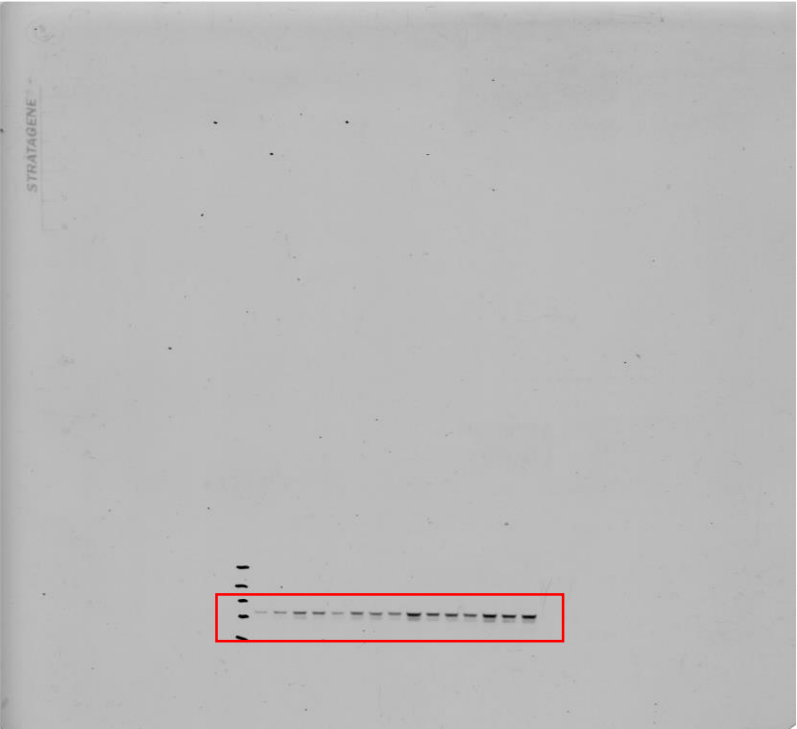

STAT3

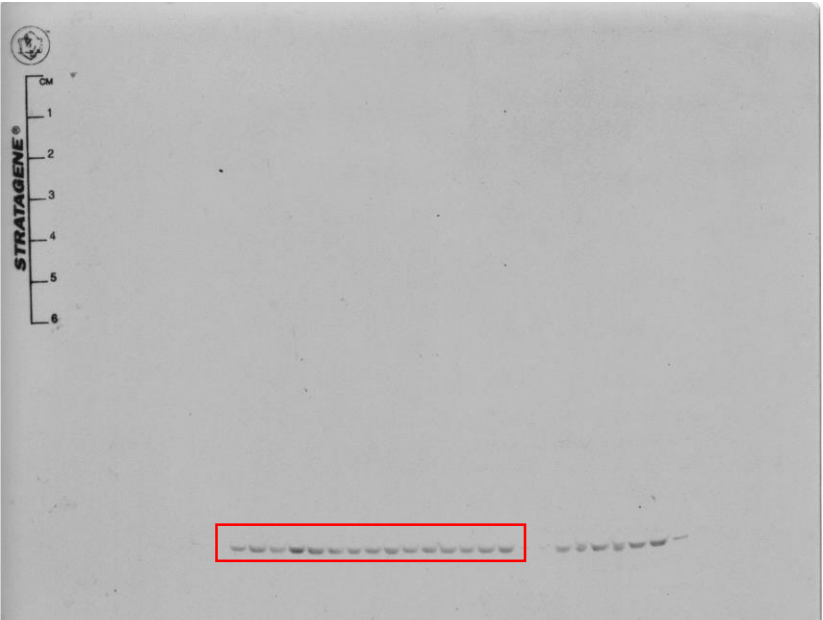

GAPDH

Supplement: Supplementary file 3 — Source Data for Expanded View and Appendix [file EMMM-12-e9910-s004.zip › EV-Appendix-SD/Source_Data_EV4G.pdf]

Appendix Figure S3B

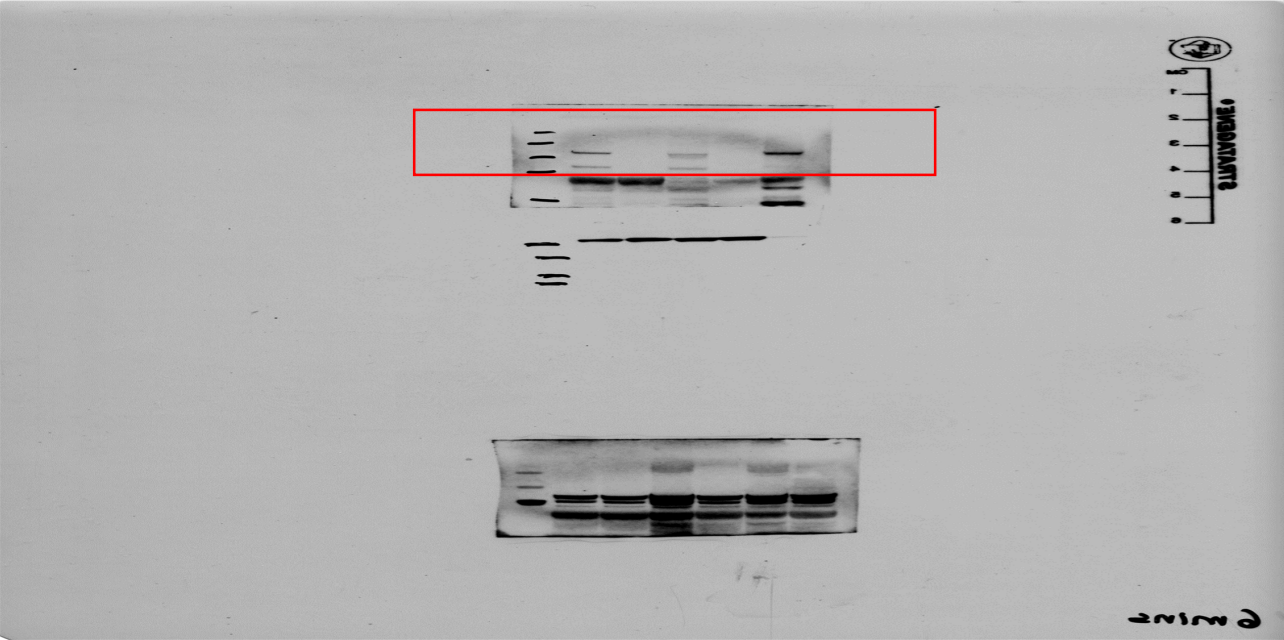

AR

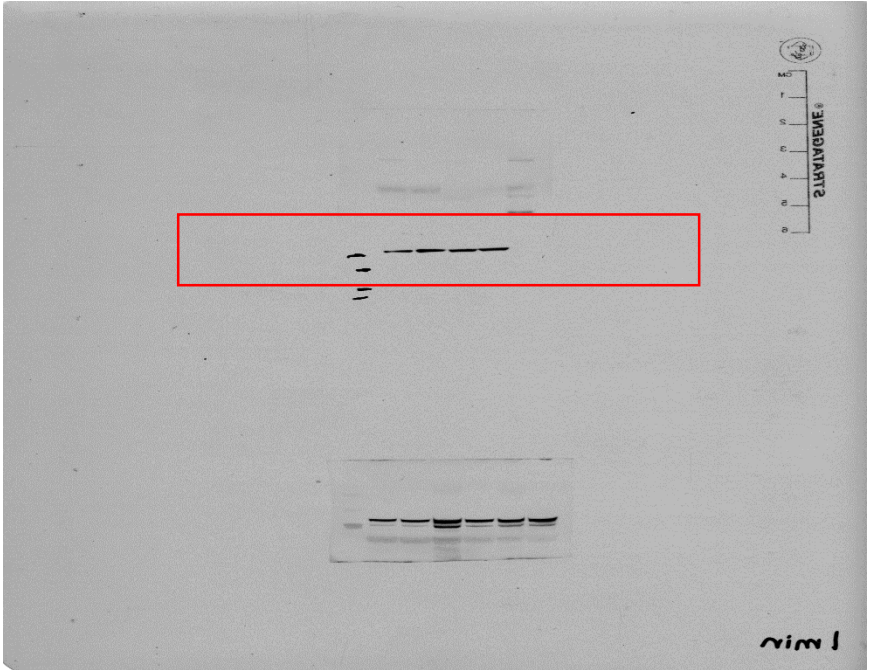

GAPDH

Supplement: Supplementary file 3 — Source Data for Expanded View and Appendix [file EMMM-12-e9910-s004.zip › EV-Appendix-SD/Source_Data_S3B.pdf]

Appendix Figure S5

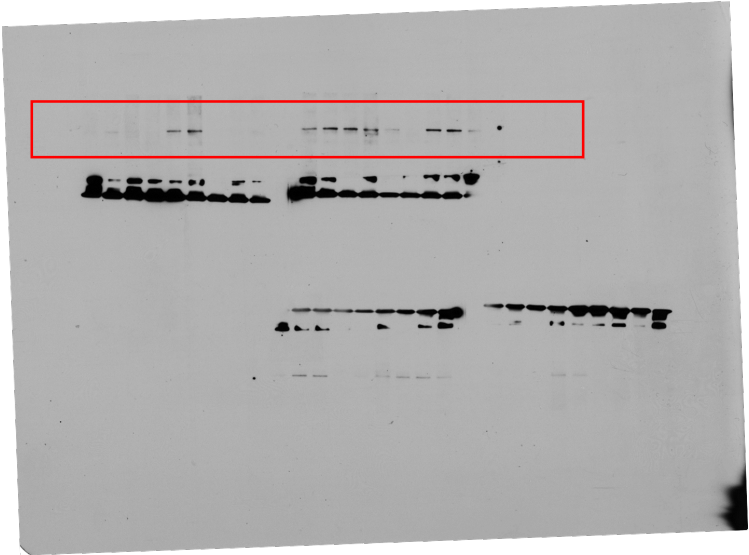

AR

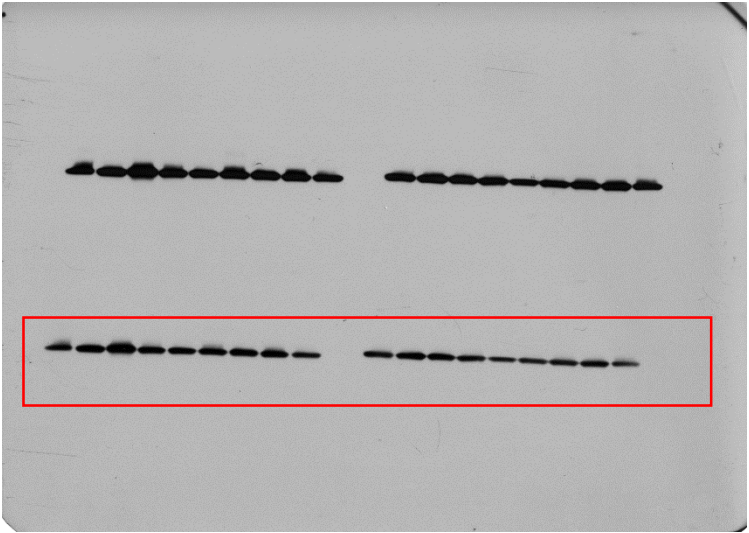

GAPDH

Supplement: Supplementary file 3 — Source Data for Expanded View and Appendix [file EMMM-12-e9910-s004.zip › EV-Appendix-SD/Source_Data_S5.pdf]

Appendix Figure S7

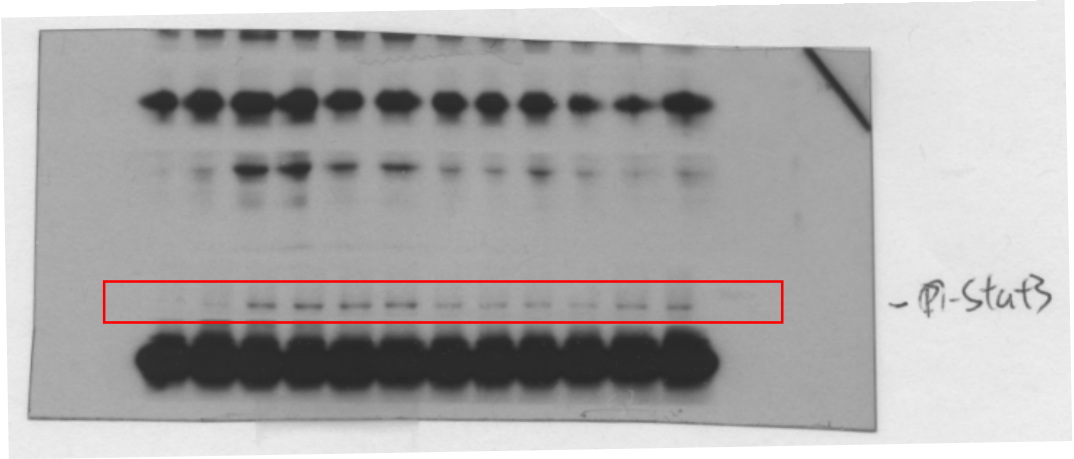

pSTAT3

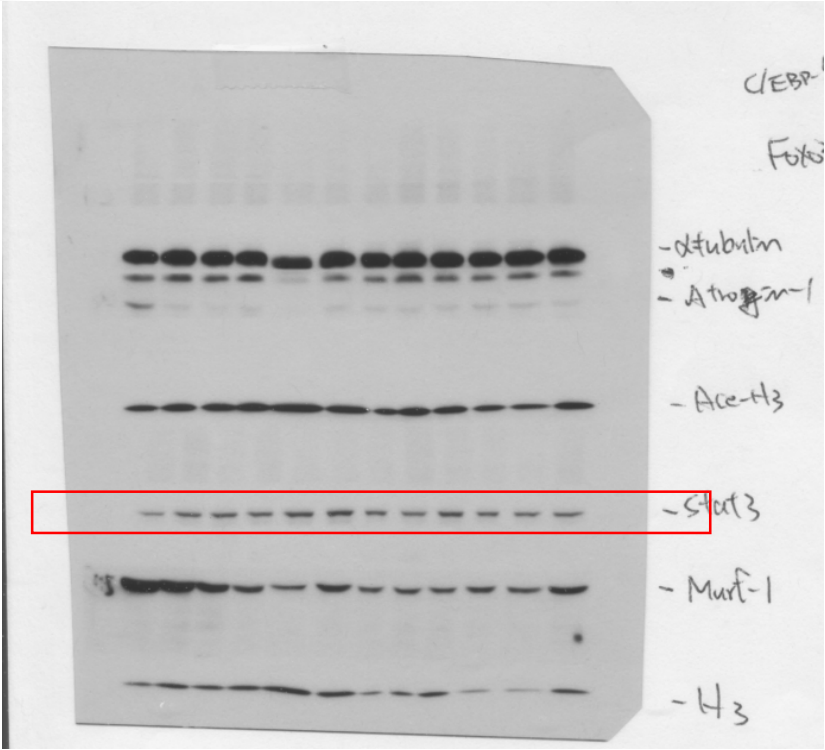

STAT3

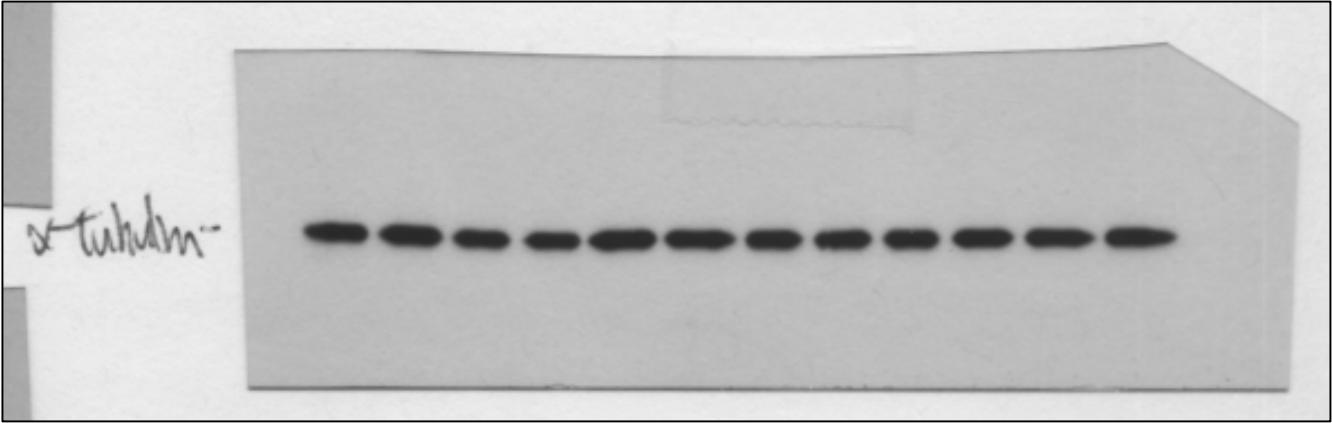

Supplement: Supplementary file 3 — Source Data for Expanded View and Appendix [file EMMM-12-e9910-s004.zip › EV-Appendix-SD/Source_Data_S7.pdf]

Figure 6B

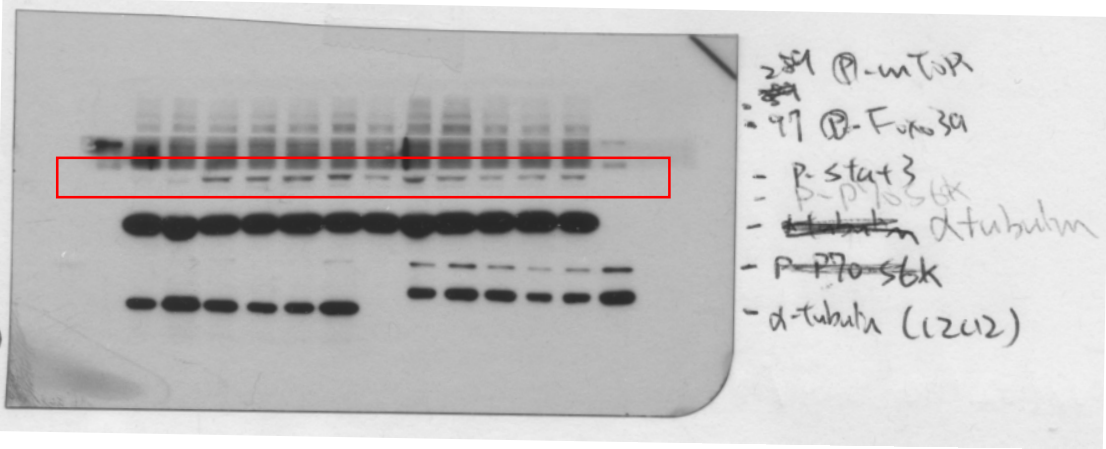

pSTAT3

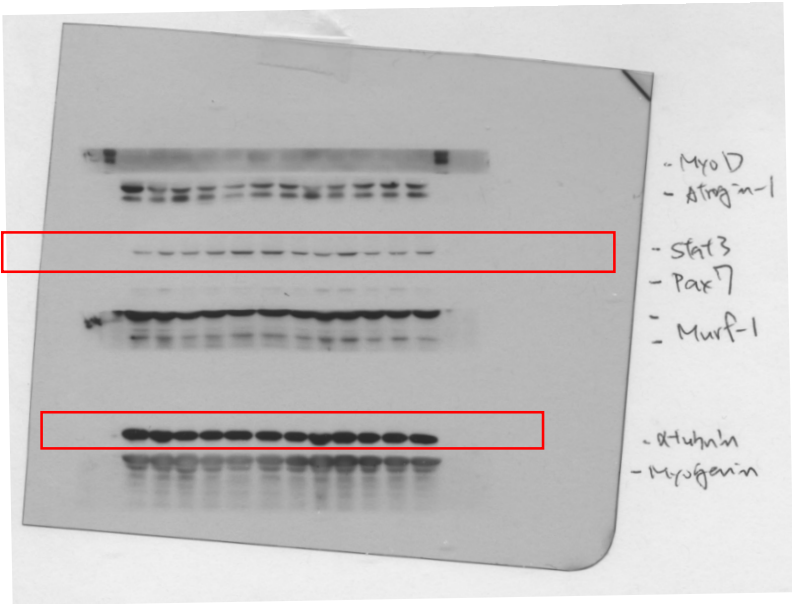

STAT3

A-tubulin

Supplement: Supplementary file 5 — Source Data for Figure 6 [file EMMM-12-e9910-s003.zip › emmm201809910-sup-0003-SDataFig6/emmm201809910-sup-0003-SDataFig6B.pdf]
