# Supplementary material for: Overcoming resistance to anabolic SARM therapy in experimental cancer cachexia with an HDAC inhibitor
Source: EMBO Mol Med. 2020 Jan 13;12(2):e9910. doi: 10.15252/emmm.201809910 (PMC7005646; doi:10.15252/emmm.201809910)
Supplement: Supplementary file 5 — Source Data for Figure 6 [file EMMM-12-e9910-s003.zip › emmm201809910-sup-0003-SDataFig6/emmm201809910-sup-0004-SDataFig6C.pdf]

Figure 6C

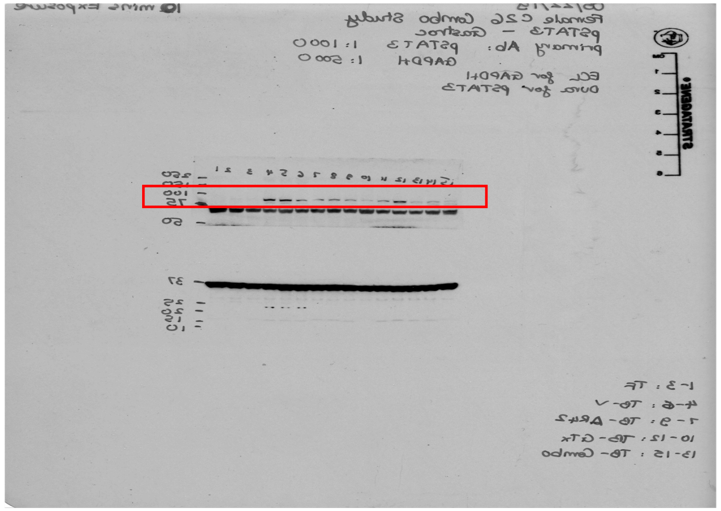

pSTAT3

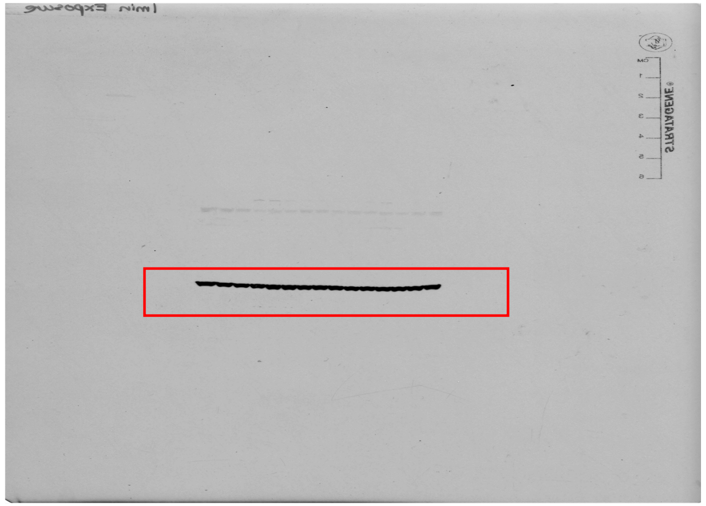

GAPDH

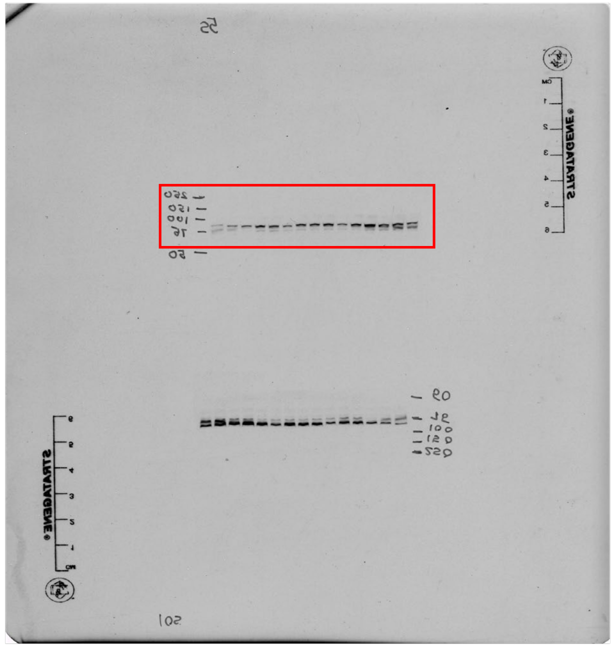

tSTAT3
